# Heavy metal contamination in groundwater: sources and sustainable remediation approaches: a review

**DOI:** 10.1039/d6ra08000f

**Published:** 2026-10-22

**Authors:** Kosar Hikmat Hama Aziz, Raz Noori Nawzad, Hemn Star

**Affiliations:** a Department of Chemistry, College of Science, University of Sulaimani Qlyasan Street Sulaymaniyah City 46001 Kurdistan Region Iraq kosar.hamaaziz@univsul.edu.iq; b Department of Earth Sciences and Petroleum, College of Science, University of Sulaimani Sulaymaniyah Iraq; c Nursing Department, Tishk International University Sulaimani Iraq

## Abstract

Groundwater has become a significant source of water for many countries. However, contamination by heavy metals has emerged as a serious global environmental issue, posing a threat to human health. Metals such as arsenic, lead, cadmium, and mercury are among the most prevalent toxins detected in groundwater, largely due to industrial activities and the effects of climate change. Heavy metals are toxic, mutagenic, carcinogenic, and prone to bioaccumulation in biological systems. Even at low exposure levels, they can damage various organs, including the nervous, cardiovascular, immune, and reproductive systems. Recent years have seen increased efforts to develop effective remediation methods. While some techniques can successfully remove heavy metal contaminants, their high operational costs may restrict their practical use. Numerous reviews have been published on the sources, toxicity, and remediation strategies for addressing heavy metal contamination in groundwater. Organic chelators like citric acid and EDTA in groundwater form complexes with heavy metals, making their removal difficult with conventional methods. Advanced oxidation processes (AOPs) are regarded as effective for decontaminating these heavy metal complexes. This review explores the sources of heavy metal contamination in groundwater, assessing their health risks and environmental impacts while highlighting recent advancements in low-cost, sustainable remediation strategies. It evaluates on-site and off-site approaches, including adsorption, ion exchange, membrane technologies, permeable reactive barriers, bioremediation, and AOPs, both as standalone and combined methods, discussing their benefits and limitations. The review also identifies research gaps, addresses current challenges, and proposes sustainable solutions for mitigating groundwater contamination.

## Abstract 2

This review critically evaluates sustainable technologies for heavy metal remediation in groundwater, comparing efficiency, environmental impacts, and practical applicability while identifying research gaps and future directions for green, scalable solutions.

## Graphical abstract

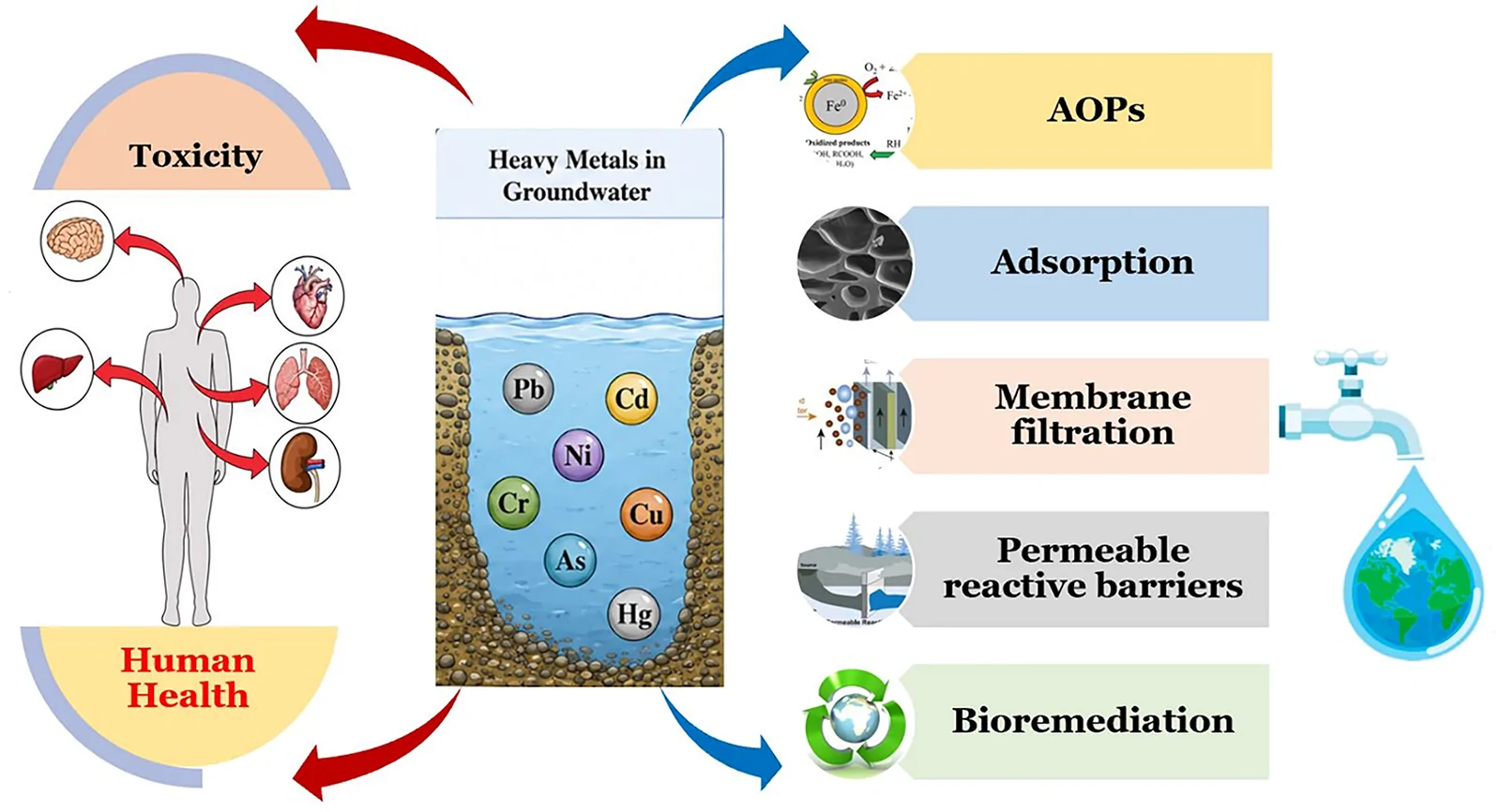

## 1. Introduction

Groundwater is important for supporting all forms of life on Earth. It serves as the main source for domestic and industrial water needs and is also the main resource for irrigation. To ensure the future quality of groundwater, it is essential to implement rigorous monitoring and effective management strategies to mitigate pollution from toxic heavy metals, all within a sustainable framework.^1,2^ Groundwater contamination by heavy metals is a global issue. The term “heavy metals” is often linked to pollution and associated toxicity, referring to a specific group of metallic substances. Among the most common and highly toxic heavy metals detected in groundwater are arsenic, cadmium, chromium, copper, lead, mercury, nickel, and zinc.^3^ Groundwater contamination from heavy metals poses significant concerns, originating from various industrial sources, mining activities, climate change, anthropogenic practices, agricultural methods, and natural weathering.^4^ These metals are particularly troubling due to their toxicity, persistence, and non-biodegradable nature.^5^ They can accumulate in the food chain, especially in plants and animals, making them among the most severe contaminants in water resources. Heavy metals such as arsenic, cadmium, chromium, mercury, nickel, and lead can exert harmful effects even at low concentrations and are recognized for their carcinogenic, mutagenic, and teratogenic properties.^6^ For instance, ingestion of water with high levels of lead and arsenic can lead to both chronic and acute poisoning, potentially resulting in cancer.^7^ The widespread occurrence, persistence, and potential toxicity of heavy metals in groundwater highlight the urgent need for effective and sustainable remediation strategies. Because the distribution, speciation, mobility, and bioavailability of heavy metals are strongly influenced by their sources and site-specific hydrogeochemical conditions, the selection of an appropriate remediation technology should be guided by contaminant characteristics, aquifer properties, and the identified pollution sources.^8^ Groundwater remediation strategies are broadly classified as *in situ* and *ex situ* approaches and include adsorption, ion exchange, membrane separation, permeable reactive barriers (PRBs), chemical precipitation, bioremediation, and oxidation–reduction processes. In addition, advanced oxidation processes (AOPs) have attracted increasing interest in the degradation and decomplexation of persistent heavy metal–organic complexes, thereby facilitating subsequent metal separation or recovery.^9^ However, remediation effectiveness should not be evaluated solely in terms of contaminant removal. Treatment cost, energy and chemical requirements, secondary waste generation, material availability and recyclability, long-term stability, and potential environmental impacts are equally important considerations. Accordingly, the development and application of low-cost, resource-efficient, recyclable, and multifunctional remediation technologies, supported by reliable source identification and site-specific risk assessment, are central to achieving sustainable management of heavy-metal-contaminated groundwater. Although several recent reviews have examined heavy-metal remediation in groundwater, many have primarily focused on individual contaminants, specific remediation technologies, or particular material classes. Comparatively, fewer reviews provide an integrated and up-to-date assessment linking pollution sources and source identification with metal speciation, site-specific hydrogeochemical controls, remediation performance, and sustainability considerations. This highlights the need for a comprehensive synthesis that connects contaminant characteristics and groundwater conditions with the selection, effectiveness, and sustainability of remediation strategies.^10–12^ Moreover, considerable attention has been devoted to heavy-metal removal from surface waters and wastewater, highlighting the need for a more groundwater-focused evaluation of treatment performance under complex and realistic hydrogeochemical conditions.^13^ Heavy metals in groundwater occur not only as free dissolved cations such as Pb^2+^, Cd^2+^, Ni^2+^, and Cu^2+^, but also as hydrolysis products, inorganic or organic complexes, colloid-associated species, and mineral-associated forms.^14^ In addition, environmentally important elements such as arsenic and chromium may occur as anionic oxo-species, including arsenate and chromate/dichromate, whose negative charge and redox-dependent speciation require remediation strategies different from those used for conventional cationic metals.^15^ Accordingly, treatment effectiveness depends strongly on contaminant speciation and site-specific pH, redox conditions, dissolved organic matter, and mineralogy. Therefore, a comprehensive and critical review of sustainable, cost-effective, and scalable remediation approaches for groundwater is needed to distinguish the relative efficiencies, limitations, environmental footprints, and practical applicability of existing technologies and to identify pathways toward greener treatment systems. Emphasis should be placed on energy and chemical requirements, secondary waste generation, regeneration and reuse, long-term stability, scalability, and performance in real groundwater matrices, which are critical for translating laboratory-scale advances into field applications. In this context, the valorization of agro-industrial waste as low-cost feedstocks for producing adsorbents, catalysts, and membrane materials offers a promising waste-to-value strategy. Such approaches can simultaneously reduce waste disposal, lower material costs, promote resource recovery and circularity, and potentially minimize the environmental burden associated with conventional material synthesis, thereby supporting the development of more sustainable and economically viable groundwater remediation technologies.^16^ This review critically evaluates major *in situ* and *ex situ* remediation approaches for heavy-metal-contaminated groundwater, including adsorption, ion exchange, membrane separation, permeable reactive barriers, chemical treatment, bioremediation, AOPs, and hybrid systems. The analysis focuses on their removal mechanisms, treatment performance, advantages, limitations, and site-specific applicability, while considering key sustainability criteria such as cost, energy and chemical consumption, secondary waste generation, material recyclability, long-term stability, and potential for resource recovery. Attention is given to adsorption as a versatile and potentially sustainable remediation strategy, emphasizing metal-uptake mechanisms and the development of green adsorbents from bio-based and waste-derived materials, including chemically modified alternatives. Their regeneration potential, resource recovery opportunities, and environmental and economic implications are also critically assessed. For heavy metal–organic complexes, the review highlights the role of AOPs in oxidative ligand degradation and decomplexation, while recognizing that the liberated metal ions remain in the aqueous phase and therefore require subsequent separation, immobilization, or recovery. This limitation highlights the potential of coupling AOPs with complementary treatment processes to achieve both ligand destruction and effective metal removal or recovery. By relating pollution sources, contaminant speciation, groundwater hydrogeochemistry, treatment mechanisms, and sustainability, the review establishes a groundwater-specific perspective for technology selection. Finally, key knowledge gaps and emerging research directions are identified to advance efficient, low-cost, resource-efficient, and environmentally sustainable remediation of heavy-metal-contaminated groundwater.

## 2. Heavy metals in groundwater: sources and health effects

Groundwater is crucial for human life, serving agricultural irrigation, drinking water, and industrial production. The rapid increase in heavy metal pollution has become a critical environmental concern due to the growing release of these contaminants into aquatic ecosystems. Once introduced, heavy metals can accumulate in sediments, posing significant environmental risks.^17^ Their high toxicity and persistence make them particularly hazardous. Historically, heavy metal levels were relatively low, but human activities have substantially disrupted natural cycles. Industrial processes, automobile emissions, and biogeochemical cycles release heavy metals into water, air, and soil. Heavy metals in groundwater originate from a combination of natural geogenic processes and anthropogenic activities, with their occurrence and abundance varying according to geological setting, land use, hydrology, and groundwater chemistry. Natural sources include weathering and dissolution of metal-bearing minerals and rocks, geothermal processes, and water–rock interactions, which can release elements such as Fe, Mn, As, Cr, Ni, and other trace metals into groundwater. Major anthropogenic sources include mining and metallurgical activities, smelting, agricultural practices, municipal waste disposal, tanning, electroplating, and fossil-fuel-related activities, as well as landfills, underground storage tanks, wastewater, and polluted surface water, which can introduce Pb, Cd, Cr, Hg, Ni, Cu, Zn, As, and other potentially toxic elements into aquifer systems.^18^ Addressing these sources is crucial to mitigating the environmental and health risks posed by heavy metal pollution in groundwater.^19^ In many contaminated sites, the distinction between natural and anthropogenic contributions is complicated by the mobilization of geogenic metals through anthropogenic changes in pH, redox conditions, groundwater recharge, and competing solutes. The environmental behavior of these metals is further determined by their chemical form and association within the groundwater system. Heavy metals may occur as free dissolved ions, hydrolyzed species, inorganic or organic complexes, colloid-associated species, and surface- or mineral-associated forms. Their distribution among these forms is controlled by pH, redox potential, dissolved organic matter, ionic strength, competing ions, mineral composition, and groundwater residence time. Redox-sensitive elements such as As and Cr are particularly important because changes in oxidation state can substantially alter their aqueous speciation, adsorption affinity, mobility, and potential bioavailability. Accordingly, characterization of both the sources and chemical forms of heavy metals is essential for understanding their transport and persistence in groundwater and for selecting remediation strategies that are compatible with the site-specific hydrogeochemical conditions.

Fig. 1 illustrates the major sources of heavy metal contamination in groundwater and the potential human-health effects associated with exposure to contaminated groundwater, particularly where groundwater is used as a source of drinking water. Depending on the contaminant, concentration, chemical form, exposure duration, and exposure pathway, prolonged exposure to contaminated drinking water may be associated with neurological, renal, hepatic, cardiovascular, gastrointestinal, and reproductive effects, as well as carcinogenic effects for certain contaminants. However, the occurrence and severity of these effects depend strongly on dose, duration, and route of exposure, metal speciation, bioavailability, and individual susceptibility. The health effects discussed in this review are therefore presented in a general toxicological context rather than as the results of a quantitative human-health risk assessment.

**Fig. 1:**
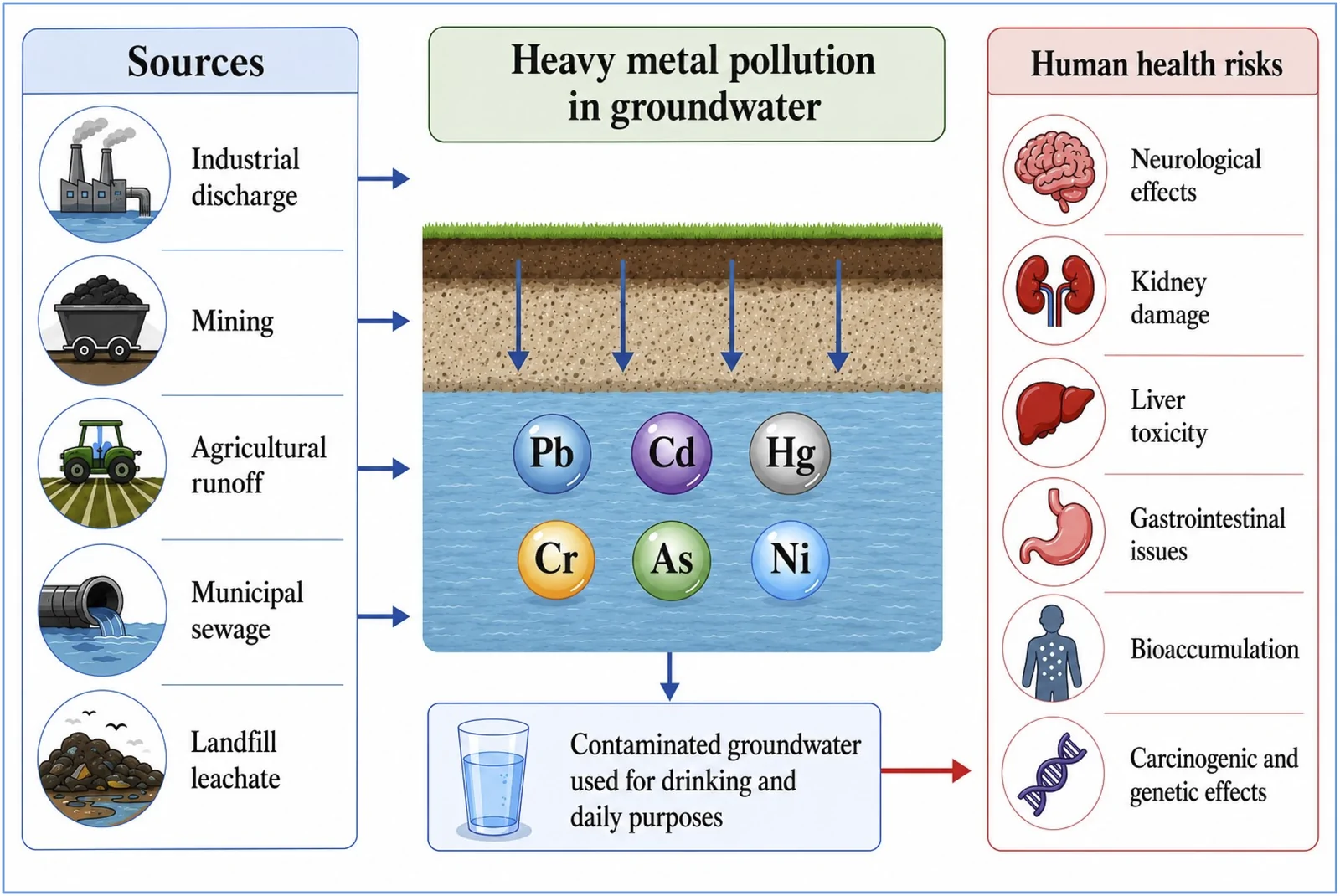
Pathways of heavy metal pollution and their health effects on the human body.

The potential health significance of heavy metal contamination highlights the importance of comparing groundwater quality with established drinking-water guidelines and regulatory values, particularly where groundwater is used for human consumption. These benchmarks provide useful reference points for interpreting measured concentrations and defining remediation targets. The World Health Organization (WHO) provides health-based guideline values, while the United States Environmental Protection Agency (USEPA) establishes maximum contaminant levels and other regulatory requirements for specific contaminants. The European Union (EU) establishes parametric values for water intended for human consumption. Because these values are developed under different regulatory frameworks and assumptions, they should be regarded as jurisdiction-specific drinking-water benchmarks rather than direct measures of individual health risk. Table 1 summarizes guidelines and regulatory values for selected toxic heavy metals and metalloids commonly encountered in groundwater.

**Table 1:** Comparison of WHO guideline values and USEPA and EU regulatory standards for selected groundwater contaminants, expressed in µg L^−1^.^20,21^

Heavy metals	WHO	USEPA	EU
Arsenic	10	10	10
Cadmium	3	5	5
Chromium	50	100	25
Lead	10	15	5
Mercury	6	2	1

Potential human-health risks from heavy-metal-contaminated groundwater can be evaluated using the hazard quotient (HQ), hazard index (HI), and carcinogenic risk (CR). HQ is calculated as the ratio of the chronic daily intake (CDI) to the corresponding reference dose (RfD), with HQ > 1 indicating a potential non-carcinogenic health concern under the assumed exposure conditions; HI is the sum of HQ values for multiple contaminants or exposure pathways, with HI > 1 indicating potential combined non-carcinogenic concern. For carcinogenic contaminants, CR is estimated from CDI and the relevant cancer slope factor and interpreted against established risk-assessment benchmarks.^22,23^ Measured groundwater concentrations should also be compared with applicable drinking-water guideline values, such as those established by the World Health Organization (WHO), to identify concentrations of potential public-health significance.^24^ In contrast, Håkanson's toxic-response factors were originally developed for ecological risk assessment of sediment-associated contaminants;^25^ therefore, their application to groundwater should be regarded as an adapted comparative screening approach rather than a direct indicator of human-health risk. Accordingly, Håkanson-based ecological risk indices should be interpreted separately from HQ, HI, and CR, which explicitly incorporate human exposure and toxicity parameters.

## 3. Identification and monitoring of contaminated groundwater

Various indexing methods have been commonly applied to evaluate heavy metal contamination in groundwater. However, multivariate chemometric techniques, such as principal component analysis and cluster analysis, are often used to address the limitations of these pollution indices. These chemometric methods are considered reliable for analyzing complex data sets, helping to differentiate between anthropogenic and natural sources of groundwater pollution.^26^ To assess groundwater quality, various indices can be utilized, including the Groundwater Quality Index (GWQI), Heavy Metal Pollution Index (HPI), Ecological Risk Index (ERI), and Contamination Index (Cd).^27^Fig. 2 illustrates the major evaluation approaches used for assessing heavy metal contamination in groundwater. Conventional pollution indices, including GWQI, HPI, ERI, and Cd, are applied to evaluate contamination levels, while chemometric techniques such as PCA and CA are utilized for reliable analysis of complex datasets and differentiation between anthropogenic and natural pollution sources.

**Fig. 2:**
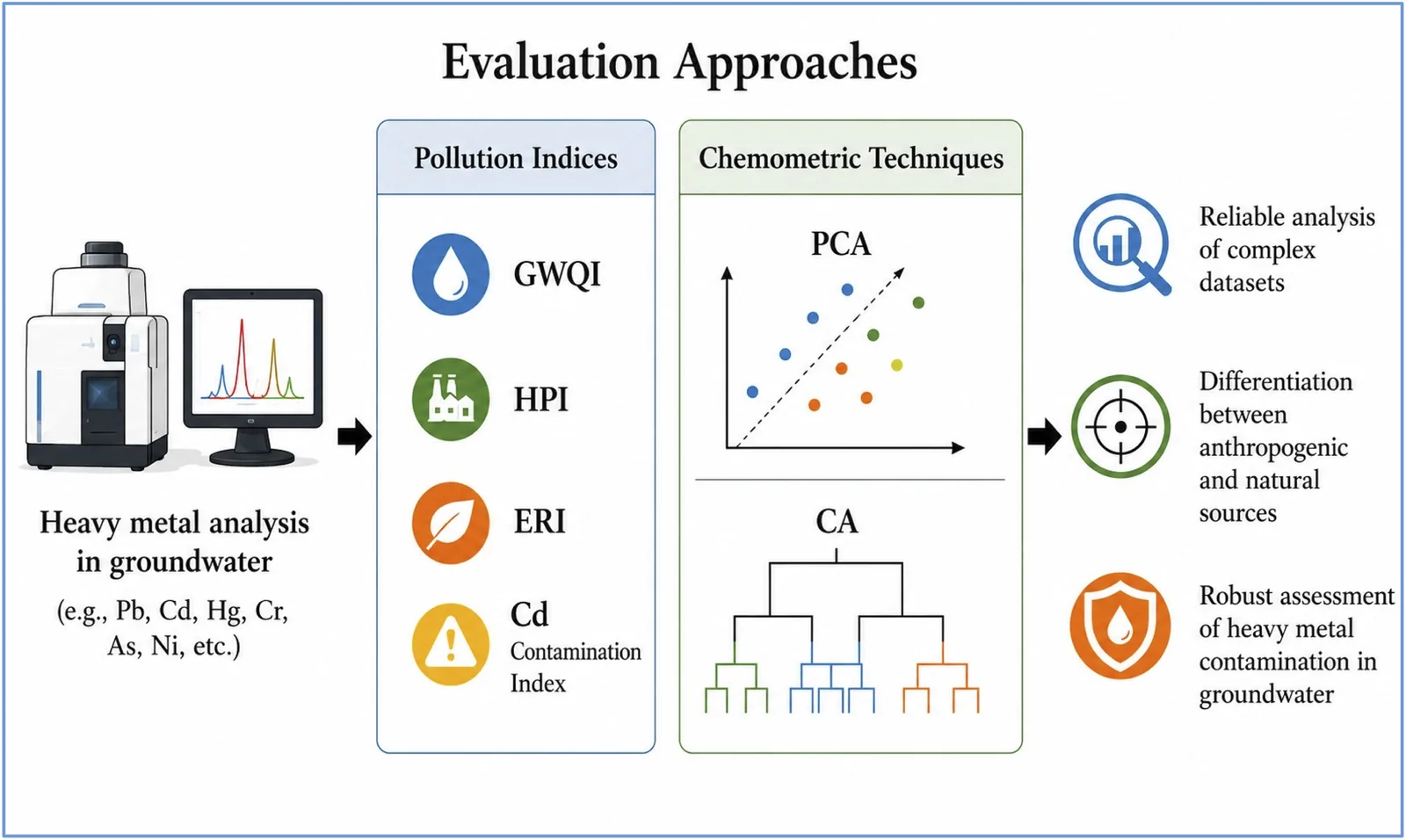
Evaluation approaches for heavy metal contamination in groundwater.

### 3.1 Sampling and analytical techniques

Rapid urbanization, mining and industrial operations, intensive farming, and inappropriate waste management are all contributing factors to groundwater contamination.^28^ Reliable assessment of groundwater quality therefore requires representative sampling strategies, appropriate analytical techniques, and rigorous quality-assurance and quality-control (QA/QC) procedures. The distribution of possible sources of pollution, well depth, land-use patterns, aquifer characteristics, and groundwater flow direction should all be reflected in the sampling locations. To differentiate natural hydrogeochemical conditions from anthropogenic inputs and assess potential contamination migration, background or upgradient wells should be monitored alongside downgradient wells wherever feasible. Sampling during both dry and wet seasons is crucial in areas with strong seasonality because changes in recharge, dilution, evaporation, and biogeochemical processes can affect groundwater chemistry and pollutant concentrations.^29^ Wells should be purged using techniques that minimize aquifer disturbance during conventional or low-flow groundwater sampling, and samples should be taken once certain field parameters, such as pH, electrical conductivity, temperature, dissolved oxygen, oxidation–reduction potential, and turbidity, have sufficiently stabilized. Consistent procedures for equipment cleaning, sample preservation, storage, and transportation are also essential to minimize cross-contamination, analyte loss, and physicochemical changes before laboratory analysis.^30^ The target contaminant and the operationally defined fraction to be measured should be taken into consideration while choosing analytical techniques. Samples are typically acidified with high-purity nitric acid to a pH of less than two after being filtered through a 0.45 µm membrane for dissolved trace-element analysis. Unfiltered samples, on the other hand, are typically used for determining total or total-recoverable elements and may need acid digestion if particulate matter is present. While atomic absorption spectrometry may be suitable when only a small number of elements are examined, ICP-MS and ICP-OES are frequently used for multi-element determination.^31^

Ion chromatography, titration, or spectrophotometric techniques are typically used to measure significant anions, while ICP-OES, atomic absorption spectrometry, or ion chromatography are frequently used to detect major cations. Depending on their physicochemical characteristics and anticipated quantities, organic pollutants are usually examined using gas chromatography or liquid chromatography, often in conjunction with mass spectrometric detection.^32^ Because contaminant mobility, bioavailability, and toxicity may depend significantly on oxidation state and chemical form rather than total concentration alone, chemical-speciation techniques, particularly chromatographic separation combined with ICP-MS, can provide valuable information on environmentally relevant contaminant forms.^33^ A systematic quality-assurance and quality-control program that includes suitable blanks, calibration verification, replicate analyses, matrix-spike or recovery tests, and certified reference materials when available should be used to establish analytical reliability. Linearity, precision, trueness or correctness, recovery, resilience, limits of detection and quantification, and measurement uncertainty should all be taken into consideration when assessing the performance of analytical methods. The integration of representative sampling, validated analytical methods, and systematic QA/QC is therefore fundamental to producing reliable, reproducible, and comparable groundwater-quality datasets for subsequent assessment and modeling.^34,35^

### 3.2 Water-quality and contamination indices

Groundwater monitoring commonly produces multidimensional datasets containing physicochemical parameters and trace-element concentrations. Consequently, index-based approaches are increasingly used to transform complex analytical information into readily interpretable numerical indicators. Water-quality indices simplify data interpretation and enable comparisons between sample locations by integrating multiple variables into a single value.^28,36^ The Groundwater Quality Index (GWQI), Heavy Metal Pollution Index (HPI), Ecological Risk Index (ERI), and Contamination Index (C_d) are some of the indicators that are frequently used. These indexes offer complementary information on cumulative contamination, possible ecological danger, trace-element pollution, and groundwater quality, respectively.^37^

#### 3.2.1 Groundwater quality index (GWQI)

The Groundwater Quality Index (GWQI) assesses whether groundwater is suitable for drinking by combining several physicochemical criteria into a single numerical number. Building on the water-quality index concept introduced by Horton (1965), the GWQI integrates multiple water-quality parameters into a single numerical indicator, thereby simplifying complex information and facilitating comparisons among sampling sites and monitoring periods.^38^

The process of developing a GWQI typically entails choosing pertinent parameters, computing quality ratings, allocating weights, combining the resulting sub-indices, and categorizing the final index value. pH, electrical conductivity, total dissolved solids, main ions, nitrate, fluoride, and certain trace elements are frequently considered. Because the selection and weighting of parameters can influence the resulting index, principal component analysis-based approaches have also been developed to identify influential parameters and reduce subjectivity in the weighting process.^39^ The weighted arithmetic water quality index (WAWQI), which is computed as follows, is one frequently used method even though there isn't a single GWQI formulation that is widely accepted:^28,39^
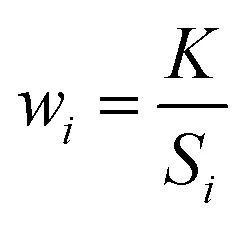

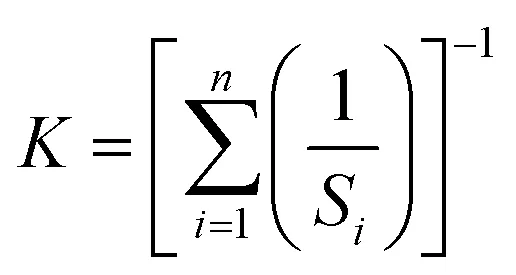

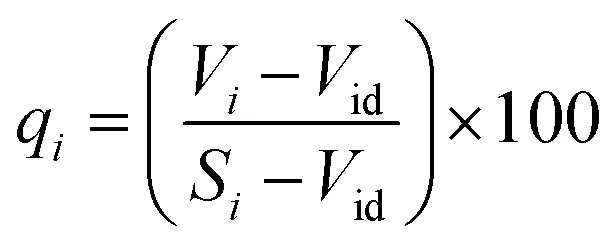

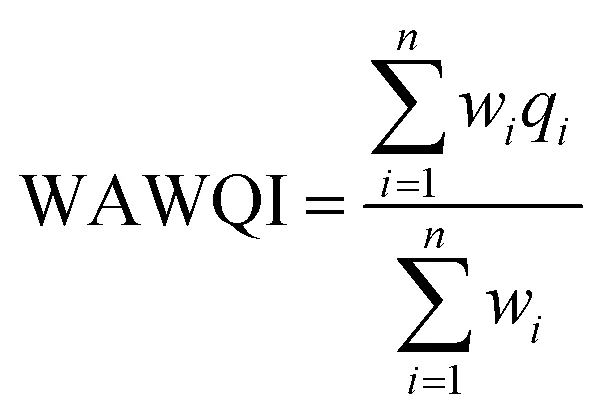
where (*w*_*i*_) stands for unit weight, (*K*) for the proportionality constant, (*S*_*i*_) for the drinking water standard, (*V*_*i*_) for the measured value, (*V*_id_) for the ideal value, (*q*_*i*_) for quality rating, and (*n*) for the number of parameters. For most metrics, zero is the optimum value, while for pH, it is 7.0.

Rapid interpretation, spatial comparison, and GIS-based mapping are all supported by GWQI. However, aggregation into a single index may conceal elevated concentrations of individual contaminants. In addition, differences in parameter selection, reference standards, weighting procedures, and classification thresholds can affect the comparability of GWQI results among studies. As a result, it should be understood in conjunction with parameter-specific findings and complementary indices like (C_d), ERI, and HPI.^28^

#### 3.2.2 Heavy metal pollution index (HPI)

The Heavy Metal Pollution Index (HPI) is one of the most widely used indices for assessing heavy metal contamination in groundwater. HPI, introduced by Mohan *et al.*,^40^ provides a straightforward and useful indicator of total heavy metal pollution by combining the concentrations of several trace elements into a single numerical value by allocating weights according to their relative relevance. HPI has been widely used for groundwater quality assessment and comparison across various hydrogeological environments due to its simplicity of calculation and understanding.^40^

The HPI is calculated as:
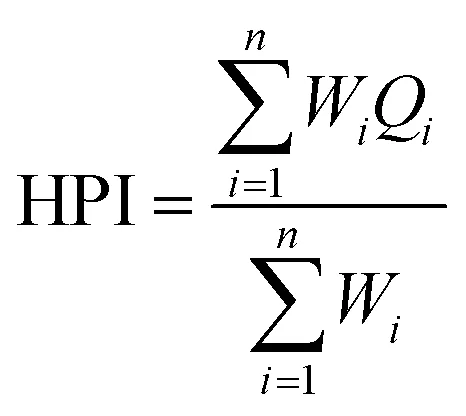
where (*Q*_*i*_) is the metal's sub-index (quality rating) and (*W*_*i*_) is the metal's unit weight. Typically, the unit weight is calculated as:
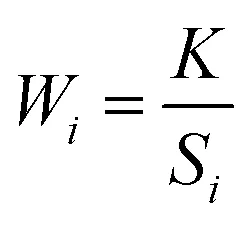
where (*S*_*i*_) is the acceptable standard value of the (*i*)th metal and (*K*) is the proportionality constant.

Trace elements like Pb, Cd, Cr, Ni, Cu, Zn, Fe, Mn, and As are frequently found in HPI. An HPI value of 100 is commonly used as a reference threshold, although the interpretation of HPI categories may vary among studies depending on the selected standards, metals, and methodological assumptions. The various toxicities of particular metals and their ecological consequences are not considered by HPI, despite its simplicity and efficacy in assessing total pollution. Therefore, HPI should preferably be interpreted together with individual metal concentrations and complementary indices such as the Heavy Metal Evaluation Index (HEI), Ecological Risk Index (ERI), and Contamination Index (C_d).^41^

#### 3.2.3 Ecological risk index (ERI)

The Potential Ecological Risk Index, commonly expressed as ERI or RI, was introduced by (Hakanson, 1980) to assess metal contamination by combining the degree of enrichment with the relative toxicity of each element. It was first created for aquatic sediments, but it has since been modified for groundwater and other environmental media to find the metals that provide the greatest ecological harm.^42^ Because the original framework was developed specifically for sediment contamination, its application to groundwater requires careful consideration of the selected reference concentrations, toxic-response factors, and environmental context.

The index is calculated as:
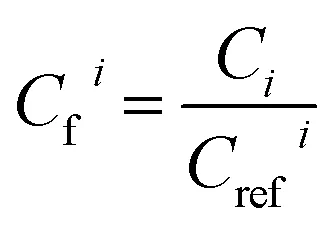
*E*_r_^*i*^ = *T*_r_^*i*^ × *C*_f_^*i*^
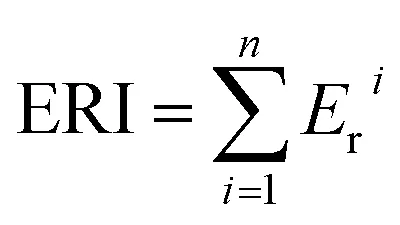
where *C*_f_^*i*^ is the contamination factor, *C*_*i*_ is the measured concentration, *C*_ref_^*i*^ is the chosen background or reference concentration, *T*_r_^*i*^ is the toxic-response factor, and *E*_r_^*i*^ is the risk related to element *i*. Hg = 40, Cd = 30, As = 10, Cu = Pb = 5, Cr = 2, and Zn = 1 are often used Hanson-based toxic-response factors. However, toxic-response factors for Ni, Mn, Fe, Co, and other elements are not consistently standardized across subsequent applications of the approach. As a result, their choice should be clearly stated and supported by the environmental medium, contaminants examined, and relevant literature.^42^

The ecological risk factor for an individual element is classified as low (less than 40), moderate (40 to less than 80), considerable (80 to less than 160), high (160 to less than 320), or very high (320 or greater). The overall ecological risk index is classified as low (less than 150), moderate (150 to less than 300), considerable (300 to less than 600), or very high (600 or greater) (Hakanson, 1980). These classification thresholds provide a standardized framework for interpreting the calculated index, but their environmental meaning depends on the applicability of the original assumptions to the investigated groundwater system. By incorporating element-specific toxic-response factors, the ecological risk index can identify significant ecological risks even when contaminant concentrations are relatively low. However, the reliability of the calculated risk is strongly influenced by the selection of background reference concentrations and toxic-response factors. Because the original index was created to evaluate metal contamination in aquatic sediments, groundwater applications should explicitly report and defend these methodological decisions.^42^

#### 3.2.4 Contamination index (C_d)

The Groundwater Contamination Index (C_d) is a widely used indicator for assessing cumulative groundwater contamination by comparing measured concentrations of water-quality parameters with their permissible limits or natural background levels. Originally proposed by Backman *et al.*,^43^ the index has been extensively applied to evaluate groundwater quality and to identify contamination associated with landfills and other potential pollution sources.^44,45^

The index is calculated as:
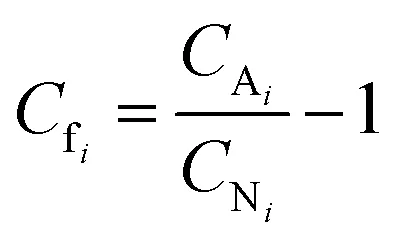

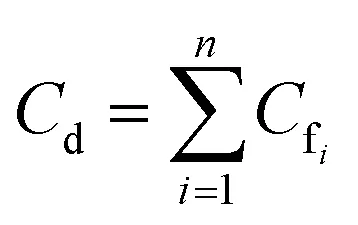
where *n* is the number of parameters, the contamination factor represents the degree of contamination for each parameter, the measured concentration is the observed concentration of each parameter, and the background or permissible concentration is the corresponding reference value. A Groundwater Contamination Index value of less than one indicates low contamination, a value between one and three indicates moderate contamination, and a value greater than three indicates high contamination.

The index facilitates the comparison of groundwater contamination in both space and time, is straightforward, and involves few computations. Nevertheless, C_d does not incorporate toxicity-based weighting, and its results can be strongly influenced by the selected parameters and by the permissible or background concentrations used as reference values. Therefore, it should be analyzed using complementary indices like HPI and ERI as well as specific parameter concentrations.

### 3.3 Geochemical and hydrogeological modeling

While water-quality indices provide useful numerical summaries of groundwater quality and contamination, they do not directly describe the hydrogeochemical processes governing contaminant occurrence, mobility, and transport. Geochemical and hydrogeological modeling can therefore complement index-based assessment by providing process-oriented interpretations of groundwater systems.

A process-based method for analyzing contamination sources, mobility, and transport in groundwater systems is offered by geochemical and hydrogeological models. Aqueous speciation, mineral-saturation states, dissolution–precipitation interactions, ion exchange, surface complexation, redox reactions, sorption, and groundwater mixing are all assessed using geochemical modeling. One-dimensional transport, batch-reaction simulations, speciation and saturation-index computations, and inverse geochemical modeling are all common uses for PHREEQC. For pollutants like arsenic, chromium, iron, and manganese, whose mobility and chemical forms are greatly impacted by pH, redox conditions, mineralogy, and sorption processes, these applications are especially crucial.^46^

Aquifer geometry, hydraulic characteristics, recharge, abstraction, and boundary conditions are all used by hydrogeological models like MODFLOW to simulate groundwater flow. They can depict advection, dispersion, molecular diffusion, sorption, mixing, mass transfer, and solute decay when combined with transport models like MT3DMS or the MODFLOW 6 Groundwater Transport model.^46^ Reactive-transport models assess the mobilization, attenuation, and persistence of contaminants by further integrating groundwater flow, solute transport, and geochemical processes.^47^

The complementary use of geochemical and hydrogeological models is particularly valuable where groundwater contamination is controlled by interactions between hydraulic transport and geochemical reactions. For example, variations in groundwater flow, recharge, redox conditions, mineral dissolution, precipitation, and sorption can collectively determine contaminant persistence and spatial distribution.

However, the conceptual model's validity, aquifer heterogeneity representation, boundary conditions, parameter estimates, and monitoring data quality all affect the model's dependability. Therefore, before using model projections to support groundwater management and cleanup decisions, calibration, sensitivity analysis, and uncertainty assessment are crucial.^48^ Model uncertainty should be explicitly considered, particularly where monitoring networks are sparse, aquifer properties are spatially heterogeneous, or geochemical reaction parameters are poorly constrained. Accordingly, integrating field measurements, water-quality indices, geochemical modeling, and hydrogeological simulations can provide a more comprehensive framework for evaluating groundwater contamination, identifying controlling processes, and supporting evidence-based groundwater management and remediation strategies.

## 4. Sustainable remediation approaches

Sustainable chemistry provides a framework for developing safer, resource-efficient, and environmentally compatible approaches to heavy-metal remediation. It prioritizes renewable or waste-derived feedstocks, safer reagents, energy-efficient processes, green catalysis, and life-cycle considerations to minimize secondary pollution and resource consumption.^49^ In groundwater remediation, these principles support the development of low-cost, recyclable, and multifunctional materials, including waste-derived adsorbents and reactive media, while promoting waste-to-value strategies and reducing treatment-related waste.^50^ Integrating sustainability into material and process design is therefore essential for advancing effective remediation technologies with lower environmental footprints and greater long-term applicability. Groundwater treatment can be performed through either on-site or off-site methods, each suited to different remediation needs. On-site treatment involves directly addressing contaminants within the aquifer, utilizing techniques such as chemical addition, utilizing the aquifer's natural mineral composition as an adsorbent, implementing membrane filtration, or using aquifer materials as catalysts in AOPs to enhance contaminant degradation or redox reactions.^51^ This *in situ* approach allows for treatment within the aquifer system, minimizing the need for groundwater extraction. Off-site treatment, on the other hand, entails extracting groundwater and transferring it to a sink reservoir or treatment facility, where it undergoes intensive processing to remove contaminants before its intended use. This *ex situ* approach provides more controlled conditions for comprehensive groundwater treatment, ensuring high-quality water is achieved for various applications. Various methods for reducing or removing heavy metals from water include adsorption, membrane filtration, zeolite ion exchange, coagulation–flocculation, chemical precipitation, AOPs, electrochemical methods, and biosorption. These techniques are broadly categorized into physical, biological, and chemical processes.^52^ Chemical coagulation–flocculation is a low-cost process but generates substantial sludge, leading to high disposal costs for large-scale use. It also has low removal efficiency and may cause secondary pollution. In contrast, electrochemical methods are highly efficient and enable selective separation without sludge production, but their high operational costs and energy requirements limit their widespread application.^53^ Membrane filtration and chemical precipitation are straightforward processes but have notable limitations. Sludge disposal can be costly, especially at large scales, and membrane fouling can reduce efficiency and increase maintenance. Additionally, chemical precipitation often requires large quantities of precipitating agents.^54^ The mentioned factors raise operational costs and pose environmental concerns, making these methods less sustainable over time. The selection of a treatment method for heavy metal removal is influenced by factors such as the type and concentration of heavy metals, regulatory limits, and the characteristics of the water.^55^ For example, organic chelators in the water can form stable heavy metal complexes that conventional methods may not effectively remove, as these methods typically target free metal ions.^56^ In contrast, AOPs are effective in decomposing these metal complexes, allowing the released free heavy metals to be subsequently removed through adsorption or ion exchange methods.^57^ An important consideration in groundwater remediation is the distinction between cationic metal species and anionic metalloids or oxyanions, because their charge, speciation, and redox state strongly control mobility, adsorption mechanisms, and treatment requirements. Cationic contaminants such as Pb^2+^, Cd^2+^, Ni^2+^, Cu^2+^, and Zn^2+^ are generally targeted through cation-exchange, electrostatic attraction, surface complexation, and chelation, for which adsorbents bearing amine, thiol, carboxyl, or other electron-donor functionalities can provide enhanced binding and selectivity. In contrast, environmentally important oxyanions such as arsenic and Cr(vi) require positively charged or metal-oxide-rich surfaces because their anionic forms interact preferentially through ligand exchange, inner-sphere complexation, electrostatic attraction, and co-precipitation. Under oxidizing conditions, As(v) occurs predominantly as H_2_AsO_4_^−^ and HAsO_4_^2−^, whereas As(iii) is mainly present as the neutral H_3_AsO_3_ species under circumneutral conditions; Cr(vi) occurs predominantly as CrO_4_^2−^ and Cr_2_O_7_^2−^, depending on pH and concentration.^58^ Consequently, iron- and manganese-oxide-based materials, layered double hydroxides, and other positively charged or metal-functionalized surfaces are particularly relevant for oxyanion removal, whereas amine- and thiol-functionalized materials are more suitable for selective capture of many cationic metals. Recent studies further demonstrate that iron-oxide sorbents can effectively target arsenic in groundwater, while manganese oxides can contribute through coupled oxidation and adsorption mechanisms.^59^ Thus, remediation-material selection should be guided by contaminant speciation rather than by total metal concentration alone, with pH and redox conditions considered as key determinants of treatment performance. These differences in charge and speciation also determine the appropriate remediation strategy. Cationic metals are commonly removed through precipitation, ion exchange, and adsorption, whereas oxoanions are preferentially treated by adsorption or ligand exchange on positively charged mineral and metal-oxide surfaces, anion exchange, co-precipitation, membrane separation, or redox transformation. For redox-sensitive contaminants such as As and Cr, speciation control is particularly important: oxidation of As(iii) to As(v) generally enhances its affinity for metal-oxide surfaces, whereas reduction of Cr(vi) to Cr(iii) can promote precipitation and immobilization. Thus, effective treatment requires consideration of pH, surface charge, competing ions, and redox conditions when selecting and designing remediation materials. Accordingly, remediation technology should be selected according to contaminant speciation and charge state, together with removal efficiency, environmental impact, treatment sustainability, and economic feasibility, to ensure effective and site-specific groundwater remediation.^60,61^ Accordingly, bioremediation, adsorption, zeolite-based ion exchange, chemical treatment, permeable reactive barriers, membrane processes, and AOPs represent complementary options for heavy metal-contaminated groundwater, each with distinct performance and sustainability characteristics. Their effectiveness depends on metal speciation, groundwater chemistry, aquifer conditions, and treatment configuration. Because reported removal efficiencies vary with experimental conditions and scale, technology selection should consider removal performance alongside treatment rate, resource requirements, secondary waste generation, long-term stability, and economic and environmental impacts. Table 2 provides a quantitative comparison of these approaches, highlighting their representative performance, mechanisms, advantages, limitations, and key factors governing their applicability.

**Table 2:** Comparison of the advantages and disadvantages of sustainable and efficient methods for heavy metal removal from groundwater.^62–64^

Methods	Efficieny	Main mechanisms	Advantages	Limitations
Bioremediation	∼60–99%	Biosorption, bioaccumulation, bioprecipitation, redox transformation	Cost-effective and environmentally sustainable; suitable for *in situ* remediation with minimal secondary pollution	Slow and condition-dependent; limited at high contamination levels; requires prolonged treatment and large land area
Adsorption	∼80–99%	Complexation, ion exchange, electrostatic attraction, precipitation, and pore filling	Efficient, cost-effective, simple, and regenerable	Requires regeneration/disposal; sensitive to competing ions and solution chemistry
Zeolite ion-exchanger	∼80–99%	Cation exchange and surface adsorption	Selective, reusable, chemically stable, and effective over a wide pH range	Competing-ion effects; chemical regeneration; limited natural-zeolite capacity
Membrane-based separation	∼90–99%	Size exclusion, electrostatic interactions, solution–diffusion, and selective transport	Selective removal; continuous, compact, and suitable for *ex situ* treatment	High energy demand; membrane fouling; metal-rich concentrate; replacement and disposal
Permeable reactive barriers	∼70–99%	Adsorption, ion exchange, precipitation, reduction, and co-precipitation	Passive *in situ* operation; low surface disturbance; adaptable media; long treatment duration	Flow- and loading-dependent; prone to clogging, media depletion, and biofouling; requires monitoring
AOPs	∼70–99% decomplexation of HMCs	Ligand oxidation, metal–ligand disruption, ROS generation, and metal separation	Rapid, effective HMC treatment; compatible with adsorption/membranes; low sludge generation	High energy and oxidant demand; scavenging and intermediate formation; requires subsequent metal separation

### 4.1 On-site (*in situ*) remediation approaches

On-site remediation of groundwater contaminated with heavy metals focuses on treating contaminants at their source to minimize environmental impact. Techniques such as permeable remediation walls, *in situ* chemical treatment, bioremediation, and soil washing and flushing are used to immobilize or remove heavy metals by transforming them, promoting uptake by plants or microbes, physical adsorption and filtration, or using chemical extraction. This approach reduces environmental disruption and mitigates the risk of groundwater pollution. However, successful implementation requires careful monitoring and site-specific optimization to ensure effective removal and long-term stability.

#### 4.1.1 Permeable remediation walls (permeable reactive barriers)

Permeable reactive barriers (PRBs) are *in situ* groundwater remediation systems that passively intercept contaminant plumes along the natural hydraulic gradient, providing an alternative to groundwater extraction and *ex situ* treatment. As contaminated groundwater flows through the reactive zone, dissolved heavy metals are removed or immobilized through adsorption, ion exchange, surface complexation, precipitation, and redox reactions, depending on the reactive medium and groundwater chemistry.^65,66^ The passive flow-through configuration generally reduces energy demand and routine operational requirements relative to pump-and-treat systems, while enabling continuous contaminant attenuation within the aquifer.^67^ However, long-term performance is controlled by reactive-media capacity, groundwater chemistry, contaminant loading, hydraulic conductivity, and preferential flow, with mineral precipitation, media passivation, and pore clogging potentially causing declining treatment efficiency and breakthrough. Thus, PRB performance should be evaluated not only by initial metal-removal efficiency but also by longevity, breakthrough behavior, hydraulic stability, and life-cycle requirements under field conditions.^68,69^ The reactive medium is a key determinant of PRB performance, and its selection should be based on the target metal, required removal pathway, groundwater chemistry, and long-term reactivity. Parameters such as pH, ionic strength, competing ions, contaminant loading, and hydraulic conductivity should be considered to ensure effective treatment without compromising groundwater flow. Among candidate materials, biochar has gained attention because of its tunable surface chemistry, porous structure, abundant functional groups, and potential for sustainable production from waste biomass, although its field performance depends on metal speciation and competitive interactions within the groundwater matrix.^70,71^ The particle size and packing of the reactive medium must be optimized to balance sufficient hydraulic conductivity with adequate contact time for contaminant removal. Excessively fine materials may increase sorption capacity but promote head loss and pore clogging, whereas coarse media can improve permeability but reduce contaminant-media contact. Therefore, hydraulic conductivity and its evolution during operation are critical design parameters for maintaining stable groundwater flow and preventing premature hydraulic failure of PRBs.^72^ A diverse range of reactive media, including zero-valent iron, zeolites, activated carbon, lime, apatite, sodium dithionite, modified red mud, metal oxides, and bio-based reactive materials, has been investigated for groundwater remediation. The choice of medium depends on contaminant speciation, groundwater chemistry, required treatment mechanism, and expected operational lifetime. Accordingly, PRBs may employ permanent, semi-permanent, or replaceable media, with replacement or regeneration typically determined by reactive-capacity depletion, passivation, contaminant breakthrough, or hydraulic deterioration.^73^ The effectiveness of PRBs is strongly influenced by metal speciation and redox conditions. For redox-sensitive contaminants, particularly Cr and As, controlling the oxidation state can substantially alter their toxicity, mobility, and affinity for the reactive medium. Accordingly, reactive amendments may be selected to promote reduction or oxidation followed by immobilization, while mild oxidants such as H_2_O_2_ can facilitate the transformation or decomplexation of metal-associated species under appropriate hydrogeochemical conditions.^74,75^ The resulting treatment efficiency therefore depends on maintaining favorable redox conditions throughout the reactive zone. To maintain groundwater flow and minimize hydraulic disruption, the reactive zone should have a hydraulic conductivity comparable to or greater than that of the surrounding aquifer. The barrier must also be appropriately keyed into the aquifer–aquitard system to minimize contaminant bypass and preferential flow, with deeper interception required where funnel-and-gate configurations are used.^76^ The regeneration, replacement, and reuse of reactive media are important for extending PRB service life and reducing life-cycle impacts. Although some sorptive media can undergo repeated regeneration, their capacity may decline because of site-specific fouling, irreversible binding, mineral precipitation, or pore blockage. Functionally, PRBs can be classified into sorption-based, chemically reactive, and biologically active systems, according to the dominant treatment process. For heavy-metal remediation, sorption-based barriers are particularly relevant because they enable passive immobilization of dissolved metals, although their long-term applicability depends on media capacity, regeneration feasibility, and breakthrough behavior.^77,78^ The reusability and resource efficiency of reactive media are important considerations for sustainable and cost-effective PRB design. Consequently, natural biopolymers, bio-based materials, and industrial wastes are increasingly investigated as alternative reactive media. Their performance can be enhanced through abundant surface functional groups that promote surface complexation, electrostatic interactions, and electron transfer, as well as through engineered core–shell or hierarchical porous structures that increase accessible reactive sites and mass transfer.^79^ PRB performance is strongly determined by aquifer hydrogeochemistry, particularly pH, dissolved oxygen, ionic strength, and competing solutes, which can alter metal speciation and the stability of metal–media interactions. Changes in pH are especially important because they influence both sorbent surface charge and metal speciation, thereby affecting retention and potential remobilization. Although sorption-based PRBs are commonly suited to relatively shallow aquifers, their long-term effectiveness depends on maintaining geochemical conditions that preserve contaminant immobilization. Changes in redox state or groundwater chemistry may destabilize retained metals and cause secondary release, highlighting the need for long-term monitoring of both contaminant concentrations and hydrogeochemical conditions.^80^ Accordingly, the suitability of reactive media should be assessed under representative groundwater conditions using batch and column experiments, with particular attention to removal capacity, hydraulic compatibility, and stability before field deployment. Chemically reactive amendments can modify local pH and redox conditions to promote metal immobilization as stable mineral phases. Commonly investigated materials include ferrous salts, lime, limestone, fly ash, phosphate amendments, Mg- and Ca-based compounds, BaCl_2_, and zero-valent metals, although their field applicability depends on contaminant speciation, groundwater chemistry, media longevity, and potential secondary impacts.^81,82^ In contrast, biological PRBs function as passive *in situ* bioreactors, in which indigenous or stimulated microorganisms transform contaminants under controlled redox conditions and suitable nutrient or electron-donor availability. Their effectiveness depends on maintaining microbial activity and favorable geochemical conditions throughout the reactive zone, making long-term performance sensitive to substrate depletion, competing electron acceptors, and changes in groundwater chemistry. Fig. 3 schematically illustrates the interception of a heavy metal-contaminated groundwater plume by a PRB and the subsequent treatment of groundwater as it passes through the reactive zone.

**Fig. 3:**
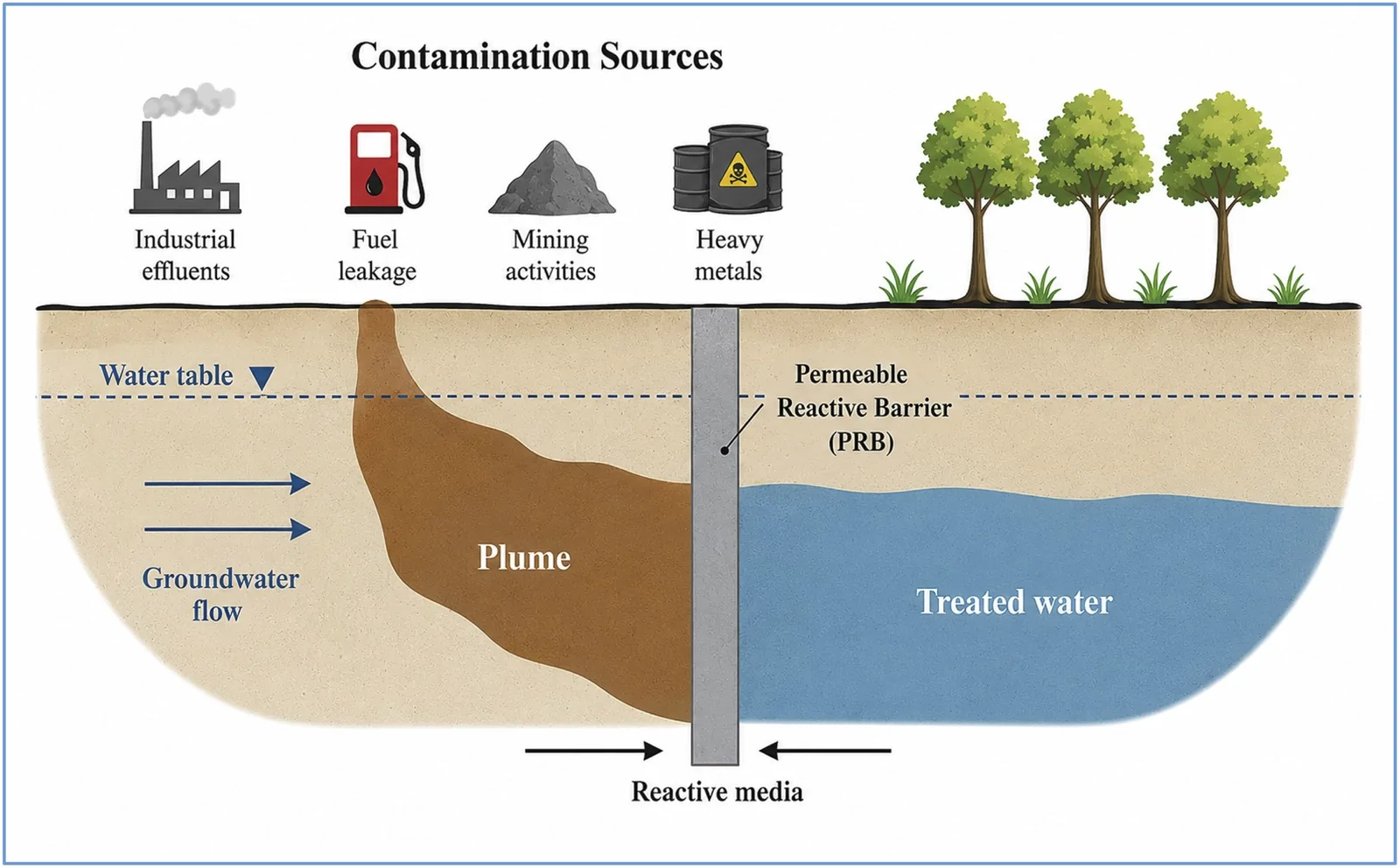
Permeable reactive barriers for heavy metal groundwater remediation, reproduced from ref. 68 with permission from [Elsevier],^68^ copyright 2026.

#### 4.1.2 Chemical treatment

Chemical treatment is an important *in situ* remediation strategy for heavy-metal-contaminated groundwater, in which reactive chemicals or amendments are introduced directly into the contaminated aquifer to transform dissolved metals into less mobile, less bioavailable, or more readily recoverable forms. Depending on the metal and hydrogeochemical conditions, treatment may involve precipitation, adsorption, complexation, mineralization, and oxidation–reduction reactions.^83^ For example, alkaline amendments can promote hydroxide or carbonate precipitation, while sulfide- and phosphate-based reagents can form sparingly soluble metal minerals and thereby reduce metal mobility. Redox-active materials, particularly zero-valent iron (ZVI) and nanoscale zero-valent iron (nZVI), can additionally promote reductive transformation, sorption, surface complexation, and co-precipitation; these mechanisms are particularly relevant for contaminants such as Cr(vi), As(v), Ni(ii), Pb(ii), and other redox-sensitive metal species.^84,85^ Recent reviews emphasize that the effectiveness of chemical amendments depends strongly on pH, redox potential, competing ions, groundwater composition, aquifer mineralogy, amendment distribution, and long-term geochemical stability. Consequently, although chemical treatment can avoid groundwater extraction and reduce disturbance to the subsurface, the potential for amendment depletion, passivation, secondary mineral formation, and subsequent remobilization of immobilized metals must be evaluated under realistic field conditions.^86^ The effectiveness of these processes depends on site-specific hydrogeochemical conditions, including pH, redox potential, competing ions, and groundwater composition. These factors can also influence reagent consumption, amendment longevity, and the formation of secondary minerals, potentially affecting long-term treatment performance and metal stability. Therefore, careful amendment selection and consideration of treatment longevity and potential secondary impacts are essential for sustainable *in situ* remediation of heavy-metal-contaminated groundwater.

#### 4.1.3 Bioremediation (*in situ*)

Bioremediation is a sustainable and environmentally friendly approach for removing heavy metals from contaminated groundwater through biological mechanisms such as immobilization, transformation, and removal of metal ions. Microorganisms contribute significantly by facilitating biosorption, bioaccumulation, biotransformation, and biomineralization processes. Phytoremediation, particularly rhizofiltration, employs plant roots to uptake, stabilize, or detoxify heavy metals directly from aqueous environments.^87^Fig. 4 illustrates the bioremediation of heavy metal-contaminated groundwater through biological mechanisms, including metal binding, metal conversion, and metal uptake. Microorganisms and plants facilitate the immobilization, transformation, and removal of heavy metal ions, resulting in improved groundwater quality. Natural biological processes alone are generally insufficient for remediating heavy metals in deep soils and aquifers. Nevertheless, phytoremediation, phytoextraction, and hyperaccumulation offer sustainable long-term remediation strategies when combined with more intensive treatment technologies, while genetically engineered microorganisms and plants can further enhance remediation efficiency. Among biological approaches, *in situ* bioremediation can immobilize or transform dissolved metals through biosorption, bioaccumulation, bioprecipitation, and microbially mediated redox reactions. Biosorption occurs through ion exchange, electrostatic interactions, and surface complexation with microbial functional groups and does not necessarily require metabolic activity.^88^ Under aerobic conditions, microbial activity can promote metal immobilization indirectly through Fe/Mn oxidation and the formation of reactive (oxy)hydroxides that adsorb or co-precipitate dissolved metals. Under anaerobic conditions, particularly where sulfate-reducing bacteria are stimulated by suitable electron donors, microbial sulfate reduction generates sulfide that precipitates dissolved metals as sparingly soluble metal sulfides, thereby promoting their immobilization.^89^ The effectiveness and stability of these processes depend strongly on pH, redox potential, electron-donor availability, competing ions, groundwater flow, and metal speciation. Accordingly, site-specific control of hydrogeochemical and microbial conditions is essential for sustained *in situ* metal immobilization.

**Fig. 4:**
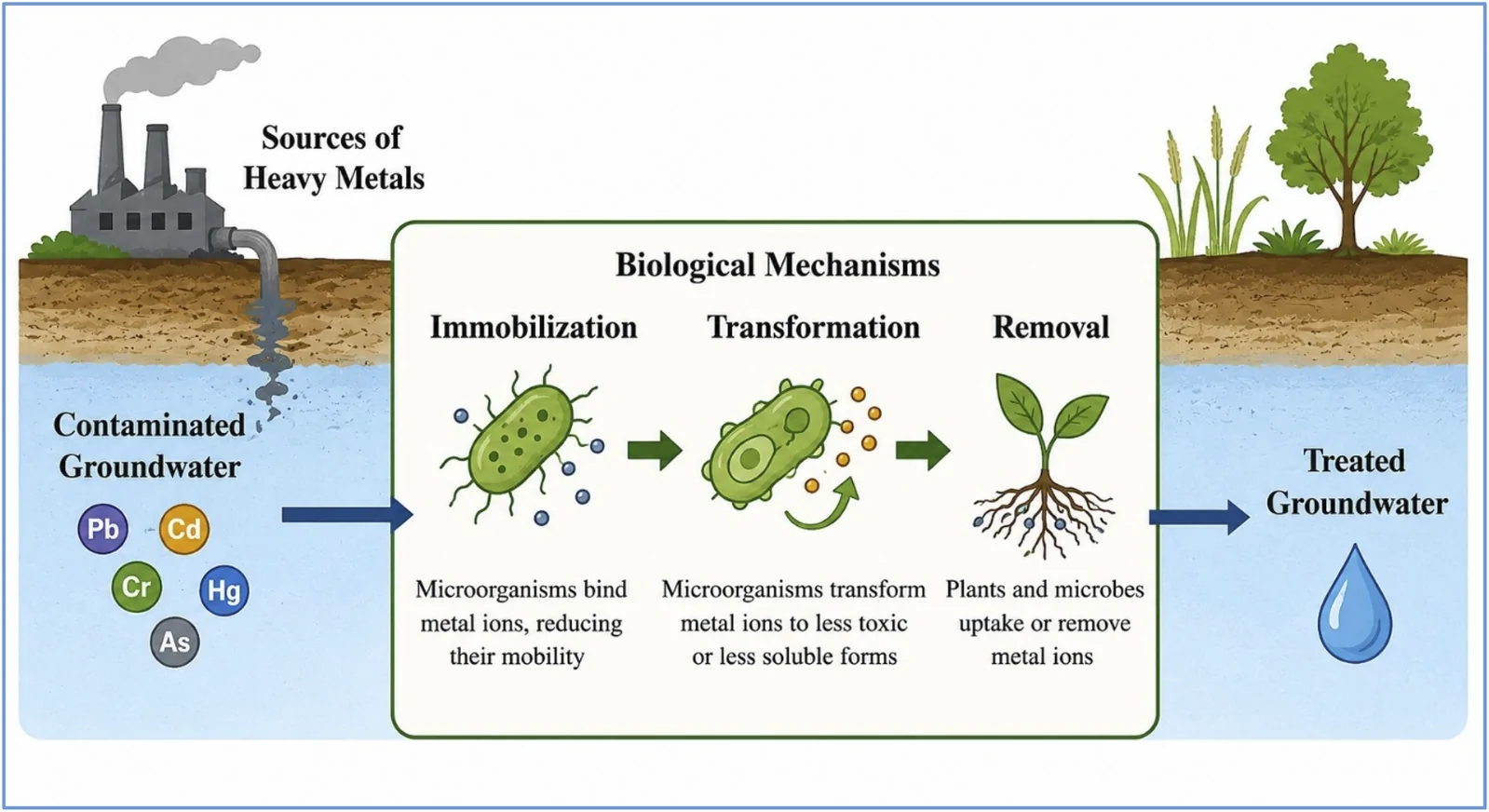
Bioremediation method for heavy metal groundwater remediation.

#### 4.1.4 Soil washing and flushing

Soil washing and soil flushing are effective techniques for removing heavy metals from contaminated soil and groundwater, functioning as *ex situ* and *in situ* methods, respectively. Both rely on washing agents such as acids, chelating agents (*e.g.*, EDTA, citric acid), surfactants, or biosurfactants like rhamnolipids to mobilize metal ions for recovery. Soil washing involves excavating the soil and treating it with liquid solutions in a controlled setting, making it suitable for coarse-textured soils and enabling the reuse of washing agents.^90^ In contrast, soil flushing applies washing solutions directly to the contaminated site to leach metals toward extraction wells, particularly in sandy or permeable soils. While effective, soil flushing requires careful monitoring to prevent contaminant migration. These approaches can be combined with other methods, such as adsorption or phytoremediation, to improve remediation efficiency and minimize environmental risks. Fig. 5 illustrates the application of soil washing and soil flushing techniques for the remediation of heavy metal-contaminated soil and groundwater. Soil washing is performed *ex situ* using washing agents to extract heavy metals from excavated soil, whereas soil flushing is conducted *in situ* by injecting washing solutions to mobilize contaminants toward extraction wells for recovery and treatment. Membrane technology can be integrated with soil washing or soil flushing to concentrate heavy metals from the washing solution, facilitating subsequent treatment or recovery of the concentrated stream while enabling reuse of the treated effluent. Common membrane systems include electrodialysis, liquid membranes, polymeric membranes, ultrafiltration, nanofiltration, and nanofiber-based membranes. Soil washing and flushing can effectively remove heavy metals from highly contaminated soil and groundwater, particularly at hotspots. However, their high water and chemical requirements, potential for secondary contamination, and disruption of soil properties limit their sustainability and large-scale application. Therefore, these methods are best suited to severely contaminated sites where conventional *in situ* approaches are insufficient.

**Fig. 5:**
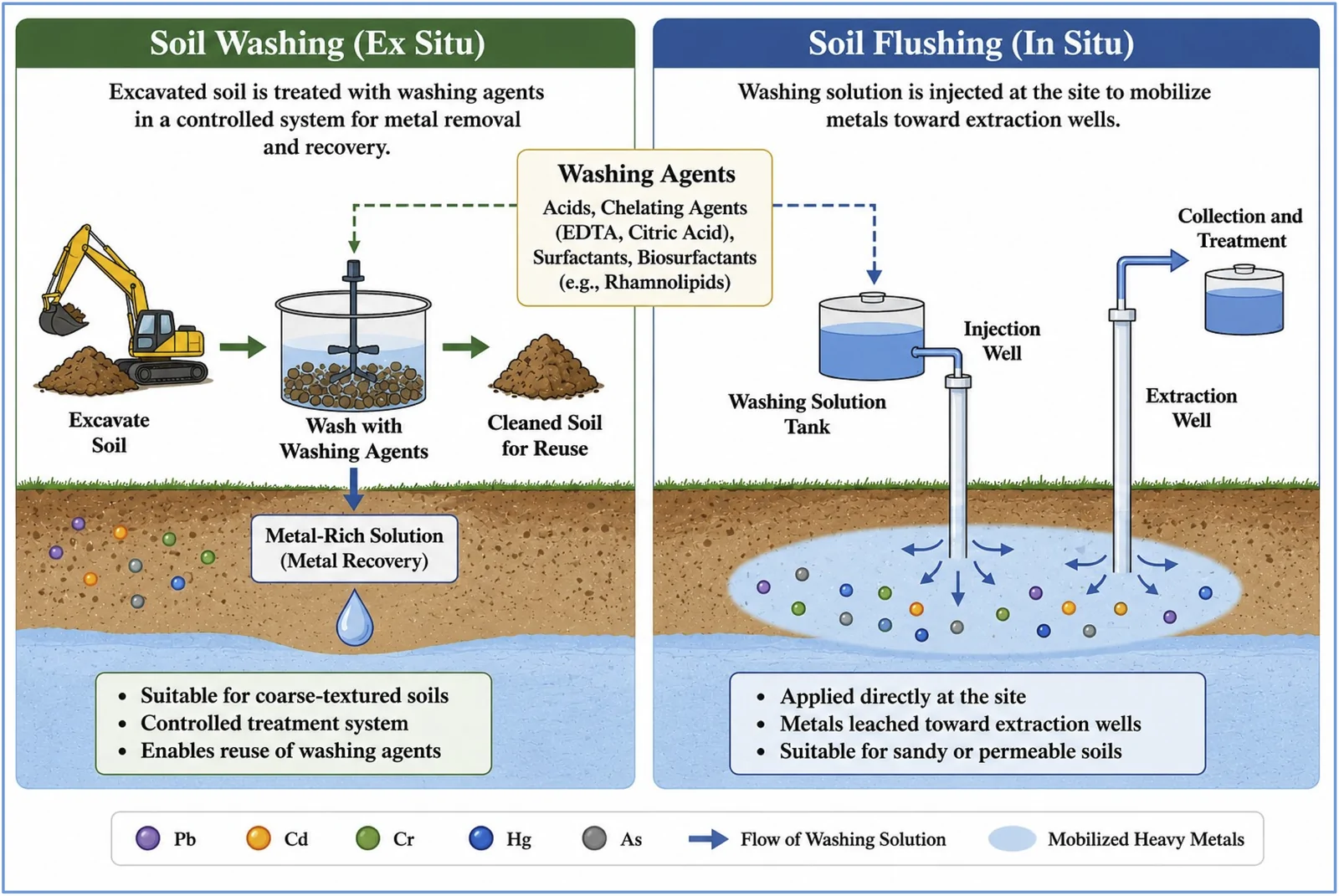
Soil washing and soil flushing for heavy metal-contaminated soil and groundwater remediation.

### 4.2 Off-site (*ex situ*) approaches

Off-site remediation approaches for heavy metal-contaminated groundwater involve the extraction of contaminated groundwater followed by its transportation to a treatment facility located outside the contaminated site. These approaches are generally classified as *ex situ* remediation because the contaminated water is removed from its original subsurface environment before treatment.^91^ Techniques such as pump-and-treat, where contaminated groundwater is pumped to the surface and treated through bioremediation, adsorption, zeolite ion-exchange, and advanced oxidation for heavy metal complex treatment as efficient and sustainable treatment approaches. Other methods include the use of membrane filtration, chemical precipitation, or reverse osmosis to remove heavy metals. While effective in reducing contaminant levels, off-site remediation is often costly, energy-intensive, and may require long-term monitoring to ensure sustained effectiveness and prevent recontamination.

#### 4.2.1 *Ex situ* bioremediation (microbial and phytoremediation)

The limitations of various remediation techniques, including high costs, toxic secondary waste generation, and significant reagent requirements for regeneration, highlight the need for alternative methods like bioremediation. This environmentally friendly approach utilizes plants and microorganisms to remove toxic heavy metals from aquatic environments.^92^ Bioremediation has demonstrated efficiency in treating polluted soils and waters, promoting sustainability and restoring the natural state of contaminated systems at a low cost, with lasting environmental benefits.^93^ However, it has limitations, such as a slow processing rate, the need for large land areas, and challenges in managing immobilized heavy metals post-treatment. Several plant species, including *Thlaspi* spp., *Urtica* spp., *Chenopodium* spp., *Polygonum sachalinense*, and *Alyssum* spp., are recognized as hyperaccumulators capable of sequestering toxic heavy metals such as Cd, Cu, Pb, Ni, and Zn. Consequently, phytoremediation offers a sustainable and cost-effective approach for mitigating heavy metal contamination in soil and indirectly improving groundwater quality through contaminant uptake and stabilization.^94^ Microbial and phytoremediation provide sustainable and environmentally benign alternatives for the remediation of heavy metal-contaminated groundwater. *Ex situ* wetland-based treatment is a cost-effective and sustainable approach for remediating heavy metal-contaminated groundwater. Through adsorption, precipitation, redox processes, and plant- and microbially mediated uptake, wetlands can effectively reduce metal concentrations while requiring relatively low energy and operational costs.^95^ Although their effectiveness may be limited by slow remediation rates and site-specific conditions, integrating bioremediation with complementary technologies can significantly enhance treatment efficiency and support long-term groundwater restoration.

#### 4.2.2 Adsorption

Adsorption is a widely applied, cost-effective approach for removing pollutants, particularly heavy metals, from contaminated water because of its operational simplicity and high removal efficiency. The process involves the accumulation of dissolved species onto a solid adsorbent through interactions with its surface until equilibrium is established.^96^ Its performance depends on operating conditions, including pH, temperature, contact time, adsorbent dosage, and pollutant concentration, as well as adsorbent properties such as surface area, pore structure, particle size, and surface functional groups.^97^ With increasing emphasis on sustainability, considerable attention has shifted toward the development of green adsorbents derived from renewable, low-cost, and waste-derived materials.^98^ These abundant and renewable resources include agricultural and industrial wastes, agro-food by-products, natural minerals, and biological biomass, which can be converted into activated carbon, biochar, engineered biochar, and biopolymer-based adsorbents for efficient heavy-metal removal.^99^ Accordingly, low-cost materials, including biochar, activated carbon, MOFs, polymer-based nanocomposites, agricultural residues, sludge-derived materials, and other carbon-based adsorbents, are increasingly employed because of their affordability and sustainability.^100,101^ Biowaste-derived adsorbents provide porous, functionalized surfaces rich in hydroxyl, carboxyl, carbonyl, and amino groups that promote heavy-metal uptake through surface interactions. Their pore networks and binding sites enhance accessible surface area and facilitate metal-ion retention.^102,103^ Heavy-metal adsorption occurs through interconnected mechanisms, including physisorption, chemisorption, ion exchange, surface complexation, electrostatic attraction, and pore filling. Physisorption involves weak intermolecular forces and is generally reversible, whereas chemisorption involves stronger chemical interactions with surface functional groups. Ion exchange and surface complexation involve the replacement of exchangeable ions and coordination with functional groups, respectively, while electrostatic attraction depends strongly on surface charge and solution pH.^104^ Pore filling contributes to uptake of highly porous materials. These mechanisms may act synergistically depending on the adsorbent and water chemistry. The porous structure and abundant surface sites of bioadsorbents facilitate metal-ion diffusion and retention through electrostatic attraction, ion exchange, surface complexation, hydrogen bonding, van der Waals interactions, and chelation.^105^ Positively charged metal ions can interact with negatively charged functional groups, while complexation involves coordination with surface ligands; chelation is a specific form of complexation that produces stable ring structures.^106^ For bio-based adsorbents, adsorption performance should be evaluated using equilibrium, kinetic, and thermodynamic parameters rather than adsorption capacity alone. Isotherm models, particularly Langmuir and Freundlich, are widely applied to describe equilibrium uptake and provide information on adsorption-site homogeneity, surface heterogeneity, and adsorbate–adsorbent interactions.^107^ Kinetic models, including pseudo-first-order, pseudo-second-order, and intraparticle-diffusion models, can help identify the relative influence of surface reaction, external mass transfer, and pore diffusion on adsorption rates.^108^ Thermodynamic parameters (Δ*G*°, Δ*H*°, and Δ*S*°) provide complementary information on adsorption feasibility, heat effects, and changes in interfacial disorders. Importantly, these parameters should be interpreted in conjunction with groundwater pH, ionic strength, competing ions, dissolved organic matter, and metal speciation, because values obtained under simplified laboratory conditions may not directly represent the performance of bio-based adsorbents in complex groundwater matrices.^109^ In groundwater, adsorption can be substantially modified by pH, ionic strength, competing ions, dissolved organic matter, alkalinity, and natural colloids, which may alter surface charge, metal speciation, and the availability of active sites.^64^ Importantly, the adsorption behavior of cationic metals (*e.g.*, Pb^2+^, Cd^2+^, Cu^2+^, and Ni^2+^) differs fundamentally from that of anionic oxyanions (*e.g.*, AsO_4_^3−^/HAsO_4_^2−^, CrO_4_^2−^, and MoO_4_^2−^). Cationic metals are commonly retained through cation exchange, electrostatic attraction to negatively charged sites, and inner- or outer-sphere surface complexation, whereas oxyanions are preferentially removed through ligand exchange, inner-sphere complexation, and electrostatic attraction to positively charged or protonated surface sites, particularly under acidic conditions.^110^ Thus, effective adsorption in groundwater requires consideration not only of adsorption capacity but also of equilibrium behavior, rate-controlling processes, thermodynamic feasibility, aqueous speciation, and matrix competition. Despite the advances in adsorbent development, activated carbon remains a widely used benchmark for heavy-metal removal.^111^ Increasing attention is being given to waste-derived adsorbents, particularly biochar produced from agro-industrial residues by pyrolysis, offering a low-cost and sustainable option for heavy-metal removal.^112^ Emerging adsorbents, including MOFs, nanocarbons, biochar, and biopolymer-based materials, offer sustainable options for heavy-metal removal. Their performance depends on the interplay between adsorbent structure and surface chemistry and metal speciation in solution (Fig. 6). Bio-based adsorbents are particularly attractive because of their renewability, low cost, and environmental compatibility.^113^ Lignocellulosic biomass, including wood and agricultural residues, is an abundant, low-cost feedstock rich in hydroxyl, carboxyl, and phenolic groups that promote heavy-metal uptake through complexation, ion exchange, and electrostatic interactions. For example, corn cobs have shown promising potential for heavy-metal adsorption.^114^ Activated carbon remains a benchmark adsorbent, while zeolites and silica-based materials offer selective ion removal. MOFs provide tunable porosity and surface chemistry, enabling high adsorption performance.^115^ Biochar offers a sustainable alternative to activated carbon, while nano-adsorbents provide high surface reactivity and abundant active sites, enhancing adsorption kinetics and removal efficiency.

**Fig. 6:**
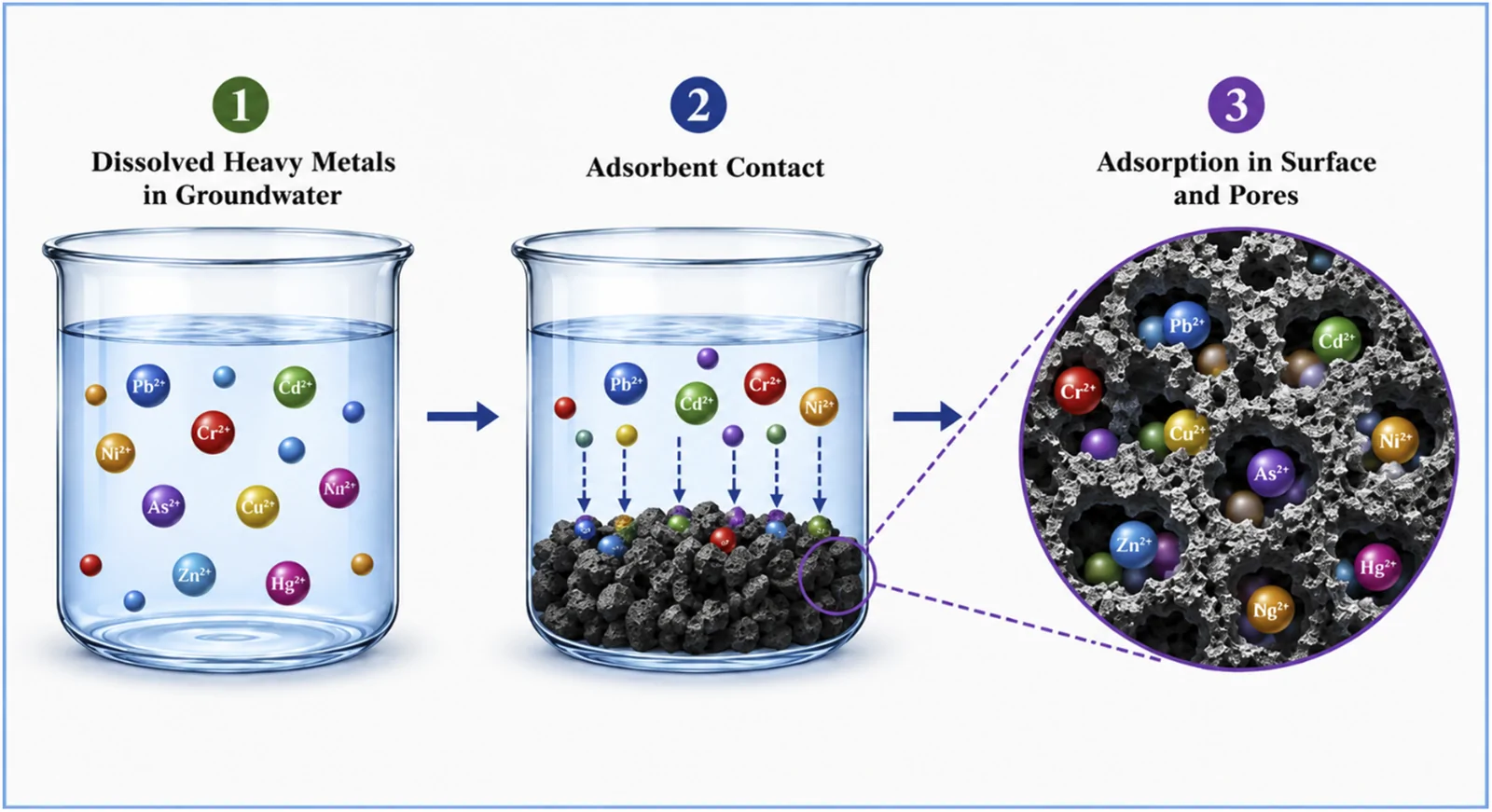
Heavy metal removal mechanism from groundwater *via* adsorption.

To provide a quantitative basis for comparison among adsorption-based remediation materials, recent studies were further evaluated in terms of adsorption capacity, removal efficiency, pH, adsorbent dosage, initial metal concentration, temperature, and water matrix (Table 3). The comparison demonstrates that adsorption performance varies substantially with both adsorbent chemistry and experimental conditions, highlighting the importance of considering operating parameters when comparing reported capacities and removal efficiencies.

**Table 3:** Quantitative adsorption performance of representative bio-based adsorbents for heavy metals and oxyanions in aqueous systems

Adsorbent	Heavy metals	Capacity	Removal (%)	pH, dose, initial concentration	Ref.
Magnetic biochar from iron-containing sludge and spent coffee grounds	Pb(ii)	181.5 µg g^−1^ from real contaminated groundwater, 40 min	86	6.0, 0.4 g L^−1^, 170.3 µg g^−1^	116
Fe-impregnated torrefied henequen biochar	As(v)	7.30 mg g^−1^, 8 h	80	pH 7.0, 2.0 g L^−1^, 1.0 mg L^−1^	117
Fe–Mn oxide-loaded sludge biochar	Cr(vi)	172.34	93	Acidic pH; dosage and concentration varied; 50 mg L^−1^	118
H_3_PO_4_-modified cocopeat biochar	Cu(ii)	566.60, 1.5 h	99	pH 5; 0.6 g L^−1^; 350 mg L^−1^	119
Magnetic biochar derived from sesame seed cake	Hg(ii)	141.89	98	pH 6; 5 g L^−1^; 30 mg L^−1^	120
Chitosan-modified magnetic biochar	Cd(ii)	149.3	80	pH 6; 2 g L^−1^; 30 mg L^−1^	121

#### 4.2.3 Zeolite-based ion exchange resin

Ion exchange represents a mature and widely adopted technology for the treatment of heavy metal-contaminated wastewater, owing to its high removal efficiency and extensive application in metal-processing and metal-finishing industries. This technique is effective for treating water with low concentrations of heavy metals. It involves the use of synthetic polymeric cation exchange resins or natural materials such as zeolites, which facilitate the exchange of metal ions. The process is based on cation exchange, where non-toxic positively charged ions, such as potassium, sodium, or hydrogen, are replaced by heavy metal ions in the solution (Fig. 7).^122,123^ Zeolite ion-exchange catalysts are widely used for heavy metal removal from wastewater and for reducing water hardness, particularly in industrial settings that utilize hot water to minimize calcium carbonate formation in pipes and boilers.^124^ This method is favored due to its rapid kinetics, high removal efficiency, minimal sludge production, and effective treatment capabilities. This process reduces sludge volume while offering additional benefits, such as selective recovery, compact treatment space, and compliance with strict discharge standards.^125^ Researchers have applied various resins for heavy metal removal using ion exchange methods. Shaidan *et al.* utilized a fixed bed containing a strong acidic cation exchange resin to remove nickel from wastewater.^126^ This resin provides several advantages, including the ability to handle large effluent volumes and more effective heavy metal removal. Furthermore, it is easily regenerated with affordable and readily available chemical reagents. The process involves directing wastewater through an ion-exchange column, where heavy metal ions are exchanged for other ions within the resin bed. When the column becomes saturated with heavy metals, backwashing is employed to remove the accumulated contaminants. Natural zeolites are excellent ion-exchangers and adsorbents for removing heavy metals, offering several advantages over synthetic materials. They are abundant, cost-effective, easily modified, and stable, with regeneration possible using low-cost reagents like NaCl.^127^ Their adsorption capacity can be enhanced through modification with metallic reagents, surfactants, acid-base treatments, and physicochemical processes. Chemical reagents, such as acids, bases, and low-cost salts like NaCl, are commonly used for the regeneration of exhausted cation exchange resins.^128^ The regeneration efficiency of potassium salts was found to be higher than that of sodium salts for all cation exchange resins. However, NaCl has several advantages as a regenerant, including high water solubility, low toxicity, and lower cost, making it more suitable for commercial applications.^129^ Despite its effectiveness in removing heavy metal ions, the use of cation exchange resins has drawbacks, particularly high operational costs, which limit its practicality for treating large volumes of wastewater with high concentrations of heavy metals. Additionally, this method is less effective for complex industrial wastewater that contains organic ligands or chelators, which form complexes with heavy metals. Natural zeolites are highly effective adsorbents for heavy metal ions, offering several advantages over synthetic ion-exchange zeolites.^130^ These benefits include efficient ion exchange, adsorption, dehydration, and rehydration capabilities, combined with low cost, easy regeneration, and wide availability. Additionally, natural zeolites can be modified through metal doping, surfactants, acids, bases, or salts to enhance their heavy metal ion-exchange capacity. Among these modifications, the most common involve increasing the native zeolite's affinity for heavy metals using metal-doping agents and surfactants.^131^ Ion exchange using natural or synthetic zeolites is among the most effective and cost-efficient technologies for removing heavy metals from contaminated groundwater. The performance of zeolites depends on their surface area, pore structure, ion-exchange capacity, and surface functional groups. Their effectiveness is commonly evaluated based on metal removal efficiency, selectivity, regeneration potential, and long-term environmental sustainability.

**Fig. 7:**
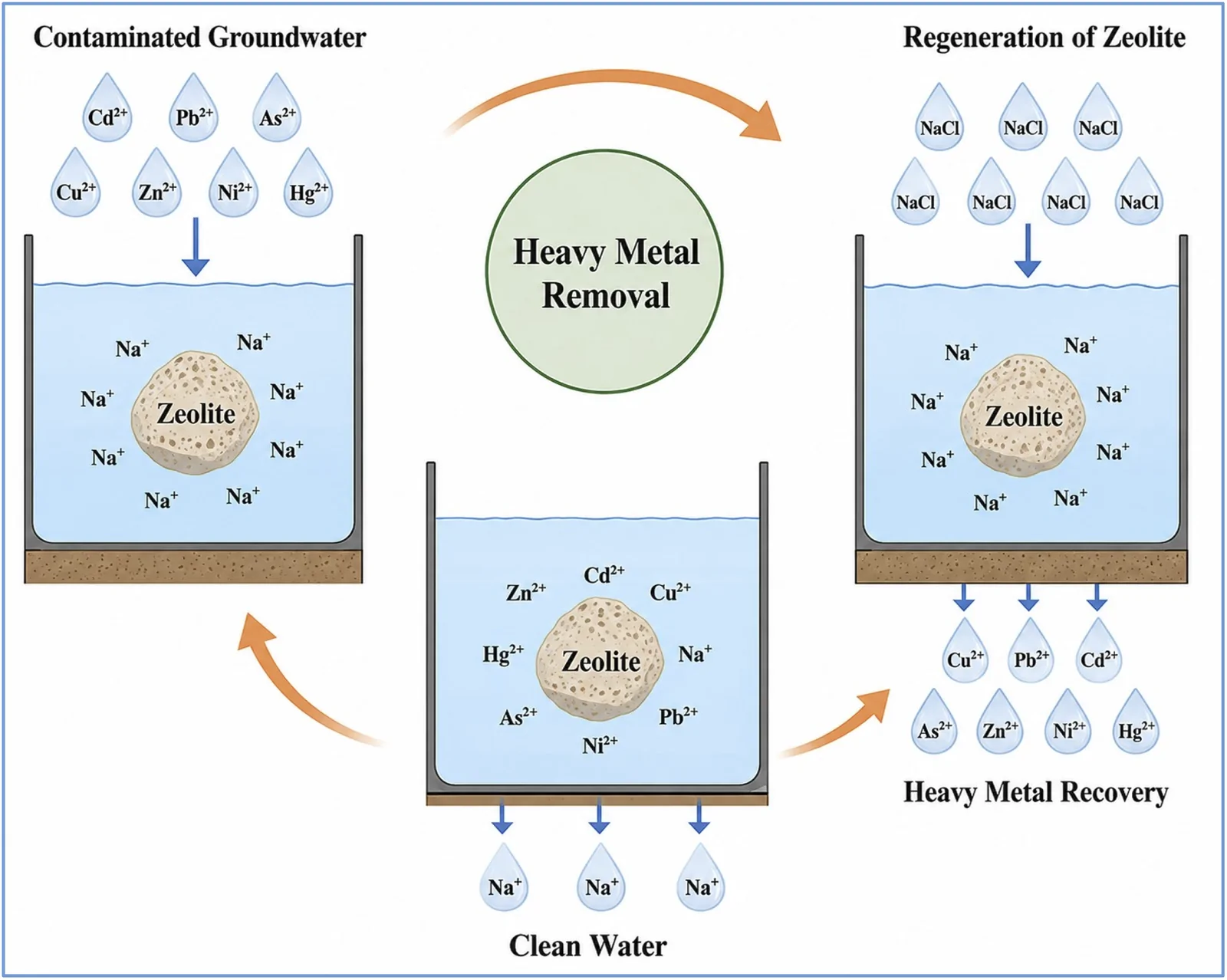
Zeolite-based ion-exchange mechanism for heavy metal remediation, reproduced from ref. 132 with permission from [Royal Society of Chemistry],^132^ copyright 2026.

#### 4.2.4 Membrane-based technology

Membrane-based technologies provide an effective *ex situ* (off-site) approach for treating heavy-metal-contaminated groundwater following extraction from the subsurface. Processes such as nanofiltration, reverse osmosis, ultrafiltration, and forward osmosis enable selective separation of dissolved metals through size exclusion, charge interactions, and other physicochemical mechanisms.^133,134^ Their performance depends on membrane characteristics, metal speciation, feed-water composition, and operating conditions. From a sustainability perspective, advances in membrane fabrication increasingly focus on low-energy operation, safer solvents and additives, durable materials, and improved fouling resistance.^135^ Nevertheless, membrane treatment can generate concentrated metal-rich streams and suffer from fouling and membrane replacement requirements; therefore, energy demand, concentrate management, membrane lifetime, and end-of-life considerations should be incorporated into the assessment of its environmental performance. Integrating these factors is essential for developing membrane-based treatment systems that are both effective and consistent with sustainable groundwater remediation. Membranes are broadly classified as isotropic or anisotropic according to their structural characteristics. Isotropic membranes possess a relatively uniform structure throughout their cross-section and may be porous or nonporous, whereas anisotropic membranes consist of distinct layers with different porosities or compositions, enabling improved separation performance.^136^ For *ex situ* groundwater treatment, membrane processes can provide continuous and selective removal of dissolved heavy metals; however, fouling, concentration polarization, and the permeability-selectivity trade-off can limit their long-term performance.^137^ Recent advances in functionalized membranes have addressed these limitations through the incorporation of highly porous and chemically stable materials.^138^ For example, incorporating UiO-66(COOH)_2_, a carboxyl-functionalized zirconium-based MOF, into PVC membranes can enhance heavy-metal removal by providing high surface area, well-defined porosity, and abundant carboxyl-containing binding sites. These functional groups facilitate interactions with metal ions, while the inherent chemical and thermal stability of UiO-66(COOH)_2_ can improve membrane durability and separation performance.^136,139^ Such MOF-functionalized membranes offer promising opportunities for developing more selective and durable membrane systems for sustainable off-site remediation of heavy-metal-contaminated groundwater.

#### 4.2.5 Advanced oxidation processes for heavy metal complexes

Advanced oxidation processes (AOPs) comprise physicochemical treatment technologies that generate highly reactive species, including hydroxyl radicals (˙OH), sulfate radicals (SO_4_˙^−^), superoxide radicals (O_2_˙^−^), and non-radical singlet oxygen (^1^O_2_), enabling the degradation and transformation of recalcitrant organic pollutants. These reactive species are generated *in situ* through the activation of oxidants such as hydrogen peroxide (H_2_O_2_), peroxymonosulfate (PMS), and persulfate (PDS) *via* thermal, photochemical, electrochemical, or homogeneous/heterogeneous catalytic pathways.^140,141^ Hydroxyl radicals are among the principal reactive oxygen species (ROS) responsible for the degradation of organic ligands coordinated to the heavy metal in complex and industrial wastewater in many AOPs. In the treatment of heavy-metal complexes, oxidative cleavage of the ligand can destabilize the metal–ligand coordination structure and progressively liberate the corresponding metal ions.^142,143^ Depending on their redox properties and the reaction environment, the released metal ions may participate in additional homogeneous catalytic or redox cycles, thereby influencing oxidant activation and ROS generation.^144^ Following decomplexation, the liberated organic ligands may undergo successive hydroxylation, bond cleavage, and oxidation, thereby converting heavy metal–organic complexes into smaller, more readily separable products and, under sufficiently strong oxidative conditions, ultimately to CO_2_, H_2_O, and inorganic ions. However, the efficiency of this AOP-based pretreatment is strongly influenced by groundwater matrix characteristics, including natural organic matter, alkalinity, inorganic ions, pH, and radical-scavenging constituents, which can consume reactive species and limit decomplexation and subsequent separation performance.^145^ The extent and selectivity of these pathways are determined by the nature of the metal–ligand bond, ligand structure, ROS reactivity, oxidant concentration, pH, and competition for ROS by background constituents. Accordingly, understanding the coupling between metal–ligand decomplexation, metal redox cycling, and ROS-mediated oxidation is critical for designing AOPs capable of effectively transforming persistent heavy-metal complexes while controlling the fate and mobility of the released metal species.^146^ AOPs include several treatment technologies, such as Fenton and photo-Fenton processes, photocatalysis, persulfate-based oxidation, ozonation, electrochemical oxidation, and non-thermal plasma.^147^ For heavy metal complexes (HMCs), AOPs can be integrated with conventional processes such as adsorption and ion exchange to facilitate ligand degradation, promote metal–ligand decomplexation, and enhance subsequent metal removal. Such hybrid strategies are particularly relevant for complex wastewaters in which stable organic ligands can limit the effectiveness of conventional metal-removal processes.^148^ HMCs form in the presence of organic chelators, such as EDTA, and other organic pollutants that tend to create stable complexes with heavy metals. These complexes are challenging to remove directly through adsorption or ion exchange,^149^ necessitating initial decomposition with strong oxidants to release heavy metal ions for easier removal by conventional methods (Fig. 8). AOPs can promote decomplexation of HMCs by generating highly reactive species, including ˙OH, SO_4_˙^−^, O_2_˙^−^, and ^1^O_2_, depending on the oxidant and activation process. Their performance is controlled by the oxidant type and dose, reactive-species yield, groundwater matrix, and extent of ligand degradation. Oxidative cleavage of metal–ligand bonds can release dissolved metal ions and convert organic ligands into smaller intermediates and, under sufficiently strong conditions, CO_2_, H_2_O, and inorganic products. However, ligand degradation alone does not constitute metal removal; the released metals must subsequently be recovered through a coupled separation step, such as adsorption, precipitation, membrane separation, or ion exchange. Thus, AOP performance should be assessed by both decomplexation efficiency and subsequent metal recovery, while minimizing excessive oxidant consumption and secondary by-product formation. Additionally, AOPs are valuable for oxidizing certain metals, such as converting As(iii) to the less toxic As(v), a safer oxidation state with reduced ecological impact. AOPs, particularly Fenton and Fenton-like systems, are effective approaches for the treatment of contaminated groundwater. Depending on aquifer characteristics, contaminant distribution, and hydrogeochemical conditions, these processes can be implemented *in situ* or *ex situ*. In iron-bearing aquifers, naturally occurring or externally introduced Fe(ii)/Fe(iii) species can activate H_2_O_2_ to generate highly reactive oxygen species, particularly hydroxyl radicals (˙OH). These reactive species can oxidatively transform organic ligands, disrupt metal–organic complexes, and thereby promote the destabilization and subsequent immobilization or removal of HMCs from groundwater. As illustrated in Fig. 8, ROS attack both the liberated organic ligands and HMCs, resulting in ligand degradation and the release of heavy metal ions. The released metals can subsequently be removed through membrane filtration, adsorption, zeolite treatment, or precipitation processes, thereby improving overall remediation efficiency.

**Fig. 8:**
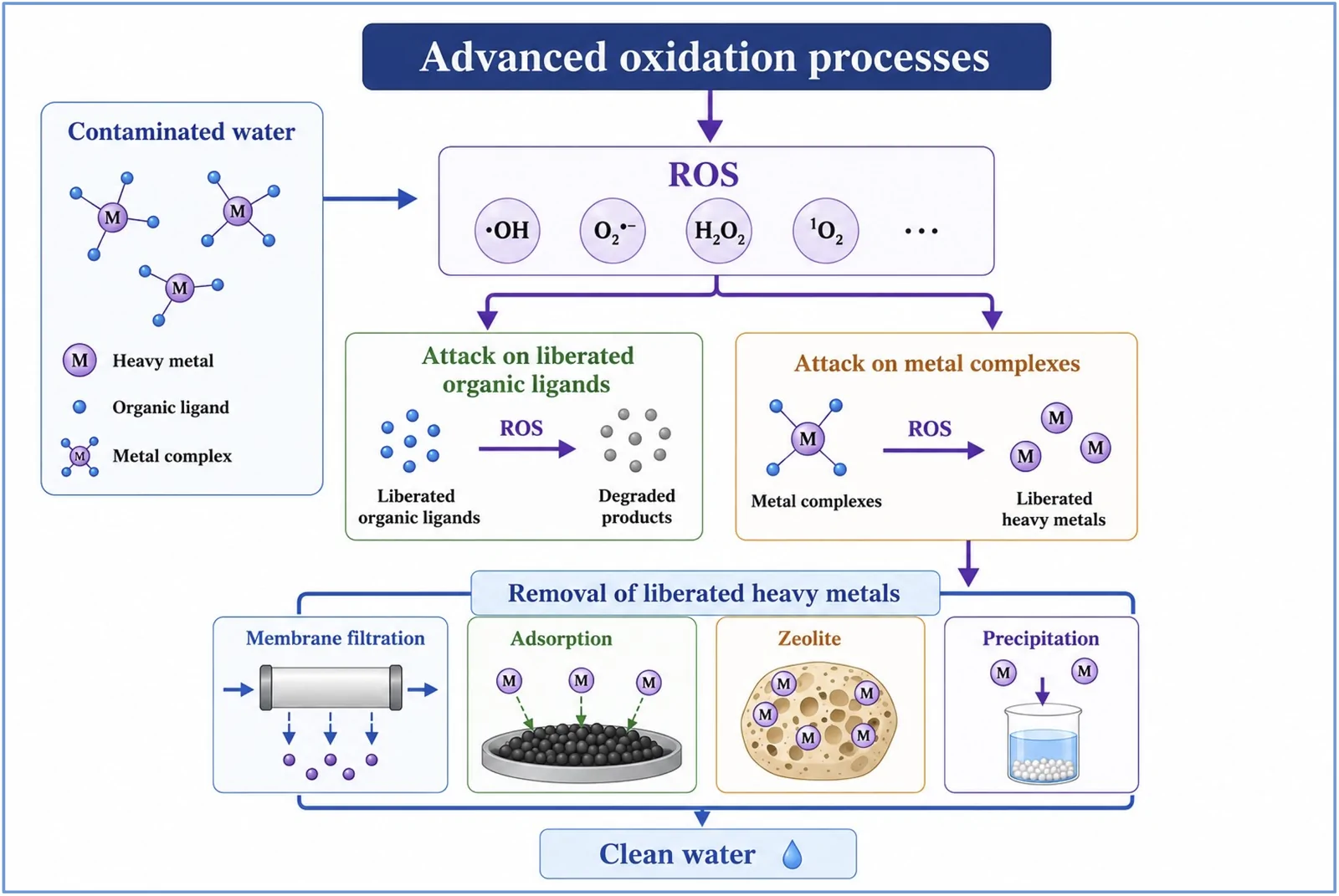
AOPs for ROS-mediated degradation of heavy metal complexes.

## 5. Challenges and limitations in remediation process

The remediation approaches discussed for heavy metal removal are both effective and sustainable. However, each method has inherent limitations that must be addressed. These challenges include varying degrees of efficiency, potential secondary waste generation, and operational costs.^150^ Further research is essential to enhance the effectiveness of these techniques and to develop strategies for overcoming their limitations. This may involve optimizing existing processes, exploring novel materials or technologies, and assessing the long-term environmental impacts of these remediation methods. By addressing these issues, we can advance the field of heavy metal remediation and improve outcomes for contaminated environments. Biobased adsorbents for removal of heavy metals from groundwater are attractive because of their low cost, abundance, biodegradability, and sustainability. However, their adsorption performance is generally lower than that of commercial activated carbon and is strongly influenced by solution conditions. Chemical modification using activating agents such as nitric acid, potassium hydroxide, zinc chloride, or phosphoric acid can significantly enhance their adsorption capacity and selectivity for heavy metal removal. Magnetic nano-adsorbents enable rapid and efficient separation using an external magnetic field, facilitating adsorbent recovery and reuse while reducing operational costs.^151^ They also exhibit high adsorption rates and enhanced selectivity toward specific contaminants. However, concerns regarding their potential ecotoxicity, long-term environmental fate, and sustainability continue to limit their large-scale application. The large-scale production of nanomaterial-based catalysts for heavy metal remediation remains challenging because of high manufacturing costs, scalability limitations, and concerns regarding the safe disposal of nanomaterial residues. Future research should therefore focus on developing low-cost, sustainable, and environmentally safe synthesis routes to facilitate their practical application.

Ion-exchange resin materials can be synthetic or natural, but studies show that synthetic resins are often preferred for their superior heavy metal ion removal efficiency. However, a notable limitation of the ion-exchange method is the production of harmful secondary pollutants from the regeneration chemicals. Additionally, this method can be expensive due to the large amounts of resin required for effective wastewater treatment and is less effective against heavy metal complexes formed by organic chelators in the water.

Bioremediation of heavy metals using plants and microorganisms is a low-cost and eco-friendly approach. However, it has limitations, including a slow processing rate, the need for large land areas, and the necessity for careful treatment and maintenance of immobilized heavy metals after the remediation process. AOPs are effective pretreatment methods for breaking down heavy metal complexes in groundwater. Organic chelators like EDTA and citric acid form stable heavy metal complexes that are challenging to remove *via* adsorption or ion exchange. AOPs can effectively decompose these complexes, enabling the removal and recovery of heavy metal ions through adsorption or zeolite ion exchange, resulting in more sustainable and efficient wastewater treatment. The success of the treatment depends on the types of heavy metals and ligands (complex stability) as well as the specific AOPs used.

## 6. Future perspectives and research directions

Despite substantial progress in understanding the sources, speciation, transport, and remediation of heavy metals in groundwater, important knowledge and implementation gaps remain across the remediation chain, from source identification to long-term treatment performance. Future research should therefore move beyond technology-specific optimization toward integrated approaches that address these interconnected challenges. A major priority is the development of standardized, long-term monitoring frameworks that combine hydrogeochemical characterization, pollution indices, isotopic fingerprinting, geospatial analysis, and machine-learning tools to improve source apportionment, predict contaminant transport, and assess temporal changes in groundwater quality. Such approaches should be coupled with improved understanding of site-specific metal speciation and hydrogeochemical controls, particularly the roles of pH, redox conditions, dissolved organic matter, competing ions, mineralogy, and groundwater flow in determining metal mobility and bioavailability.

A second critical gap concerns the translation of laboratory-scale remediation performance into reliable field applications. Although adsorption, ion exchange, PRBs, biological processes, membranes, electrochemical methods, and AOPs have demonstrated promising removal efficiencies, their performance under realistic and variable groundwater conditions remains insufficiently established.^152^ Future studies should therefore prioritize pilot- and field-scale investigations using continuous-flow systems and representative groundwater matrices, with systematic evaluation of hydraulic performance, treatment longevity, regeneration, amendment depletion, fouling, passivation, secondary mineral formation, and potential remobilization of immobilized metals. For HMCs and redox-sensitive oxoanions such as As and Cr, greater attention is also needed to speciation-dependent treatment pathways, including oxidative decomplexation, reductive transformation, selective adsorption, and subsequent metal recovery.^153^ A further knowledge gap involves the long-term environmental safety and sustainability of emerging remediation materials. Biochar and engineered waste-derived materials offer considerable potential because of their tunable surface chemistry, porous structures, low-cost feedstocks, and opportunities for waste valorization.^154^ However, future research should move beyond high adsorption capacities to evaluate regeneration efficiency, structural stability, contaminant recovery, resistance to competing groundwater constituents, and the potential release of metals, nanoparticles, and other secondary contaminants during long-term operation. Material development should prioritize performance under realistic groundwater conditions rather than equilibrium adsorption capacity alone.^155^ Finally, the sustainability of groundwater remediation should be evaluated using life-cycle assessment, techno–economic analysis, energy and carbon-footprint assessments, and resource-recovery metrics. Attention should be given to the environmental and economic consequences of chemical consumption, external energy demand, concentrate and spent material management, and repeated regeneration. Rather than assuming that hybrid or energy-intensive systems are inherently sustainable, future research should determine whether process integration, renewable energy, waste-derived materials, and resource recovery can deliver measurable reductions in environmental impacts and operating costs. Stronger collaboration among researchers, water authorities, industry, and policymakers is also required to establish standardized performance criteria and facilitate technology transfer. Overall, the key research priority is not simply to develop more efficient remediation technologies, but to establish evidence-based, site-specific treatment systems that remain effective, safe, economically viable, and environmentally sustainable over extended field operation.

## 7. Conclusions

The presence of heavy metals in groundwater is a global concern due to their toxic effects on human health and aquatic ecosystems. This review examines the main sources of heavy metal pollution in groundwater and highlights key analytical tools for monitoring their concentrations. It also discusses sustainable remediation approaches, including *in situ* and *ex situ* methods such as bioremediation, adsorption with low-cost adsorbents, permeable remediation walls, ion exchange, physicochemical treatments, and AOPs. Hybrid treatment systems integrating biochar-based adsorption and natural zeolite ion exchange with AOPs represent a promising and sustainable approach for the concurrent sequestration of heavy metals and oxidative degradation of metal–organic complexes, thereby enhancing overall remediation efficiency. However, further research is needed to enhance their performance and feasibility for large-scale, real-world applications. Biochar and natural zeolites are particularly attractive due to their low cost, reusability, ease of regeneration, and availability. Their performance can be enhanced through modifications with metal doping, surfactants, or chemical reagents to improve heavy metal removal capacity. While bioremediation offers a cost-effective option, its slow remediation rates and large land requirements are limitations. Adsorption, particularly using biochar or its modified forms, has gained attention for its high surface area and efficiency in removing diverse pollutants, providing an economical and environmentally friendly solution. Combining bioremediation with adsorption could enhance overall remediation outcomes. For large-scale applications, it is critical to prioritize the use of cost-effective biomass raw materials and non-toxic eluting agents to ensure economic feasibility and environmental safety. A major barrier to the long-term industrial application of catalysts is the progressive loss of activity caused by surface poisoning, inorganic scaling, metal leaching, structural degradation, pore blockage, and fouling by natural organic matter and other macromolecules. Future research should focus on developing robust, multifunctional, and self-cleaning catalytic membranes and adsorbents that also serve as efficient AOP activators. Such integrated materials should promote localized reactive species generation, resist fouling, maintain structural integrity, and sustain high catalytic performance under realistic operating conditions. The complexity of soil chemistry and hydrogeological conditions requires site-specific characterization to optimize remediation performance. Soil washing, chelate flushing, and physical separation may disturb soil structure and are therefore best suited for severely contaminated sites. Although *in situ* chemical injection is an effective remediation approach, it can alter aquifer geochemistry, and some reagents may be persistent or generate toxic by-products. Consequently, careful selection and controlled application of chemical amendments are essential to minimize environmental impacts.

## Conflicts of interest

The authors have no conflicts of interest to declare.

## Data Availability

No primary research results, software or code have been included and no new data were generated or analysed as part of this review.
